# Roles of TREM2 in Alzheimer’s disease

**DOI:** 10.1186/s40035-025-00515-3

**Published:** 2025-10-31

**Authors:** Xiaoshan Qi, Kedong Zhu, Wei Ke, Junjie Wang, Shanping Mao, Guiqin Chen

**Affiliations:** 1 Department of Neurology, Renmin Hospital of Wuhan University, Wuhan, 430060 China; 2 School of Physical Education, China University of Geosciences, Wuhan, 430074 China

**Keywords:** Alzheimer’s disease, TREM2, Soluble TREM2, Microglia, Amyloid, Tau, Variants, Therapeutic prospects

## Abstract

Variants in the triggering receptor expressed on myeloid cells 2 (*TREM2*) gene have been demonstrated to increase the risk of late-onset Alzheimer’s disease (AD) and Nasu-Hakola disease. As a type I transmembrane receptor, TREM2 is predominantly expressed in microglia within the central nervous system. Extensive research over the past decade has consistently established the critical role of TREM2 in AD pathogenesis, encompassing its regulation of microglial inflammatory responses, amyloid-β deposition, and tau pathology. Notably, the soluble TREM2 fragment (sTREM2) is emerging as a promising candidate biomarker for clinical progression of AD, as evidenced by human studies. Despite these advances, the precise roles of membrane-bound TREM2 and sTREM2 in AD pathogenesis remain incompletely elucidated. Novel mouse models and technological innovations have enabled therapeutic approaches targeting TREM2 for neuroprotection. This review summarizes this progress and highlights areas for future research towards the development of TREM2-directed therapeutics.

## Introduction

Alzheimer’s disease (AD) clinically manifests as memory impairment, deficits in abstract reasoning and calculation abilities, and alterations in personality or behavior [1]. As a progressive neurodegenerative disorder, the pathological processes of AD may commence decades prior to the emergence of clinical symptoms [2]. Although the initial pathogenic mechanisms remain incompletely understood, established hallmarks of AD pathogenesis include extracellular amyloid-β (Aβ) plaques and intracellular hyperphosphorylated tau neurofibrillary tangles (NFTs), alongside synaptic loss and neuronal death [3]. These pathological transformations are partially driven by neuroinflammatory cascades, now recognized as pivotal contributors to AD progression [4]. Neuroinflammation involves complex interactions among diverse cell types, with microglial activation representing a critical component of this response [4]. Triggering receptor expressed on myeloid cells 2 (TREM2), a critical receptor expressed on microglia, not only modulates neuroinflammatory responses but also regulates multiple cellular processes, including cytoskeletal reorganization, metabolic adaptation, cell survival maintenance, calcium signaling transduction, and synaptic pruning [5–9].

In 2013, two landmark genome-wide association studies (GWAS) first revealed a significant association between *TREM2* and AD. Subsequent research demonstrated that specific *TREM2* variants (e.g., R47H) are associated with a ~ threefold increased risk of developing AD compared to non-carriers [10, 11]. Over the subsequent decade, investigators have made significant advances in elucidating the biochemical properties, pathophysiological mechanisms, and therapeutic potential of TREM2. In this review, we will first summarize the biochemical properties of TREM2, then analyze the roles of TREM2, its soluble form (sTREM2), and *TREM2* genetic variants in AD pathogenesis. Finally, we will integrate recent advancements in TREM2-targeting therapeutic strategies for AD and propose directions for future investigation.

## Biochemical characteristics of TREM2

### TREM2 gene transcription and subcellular localization

**Fig. 1:**
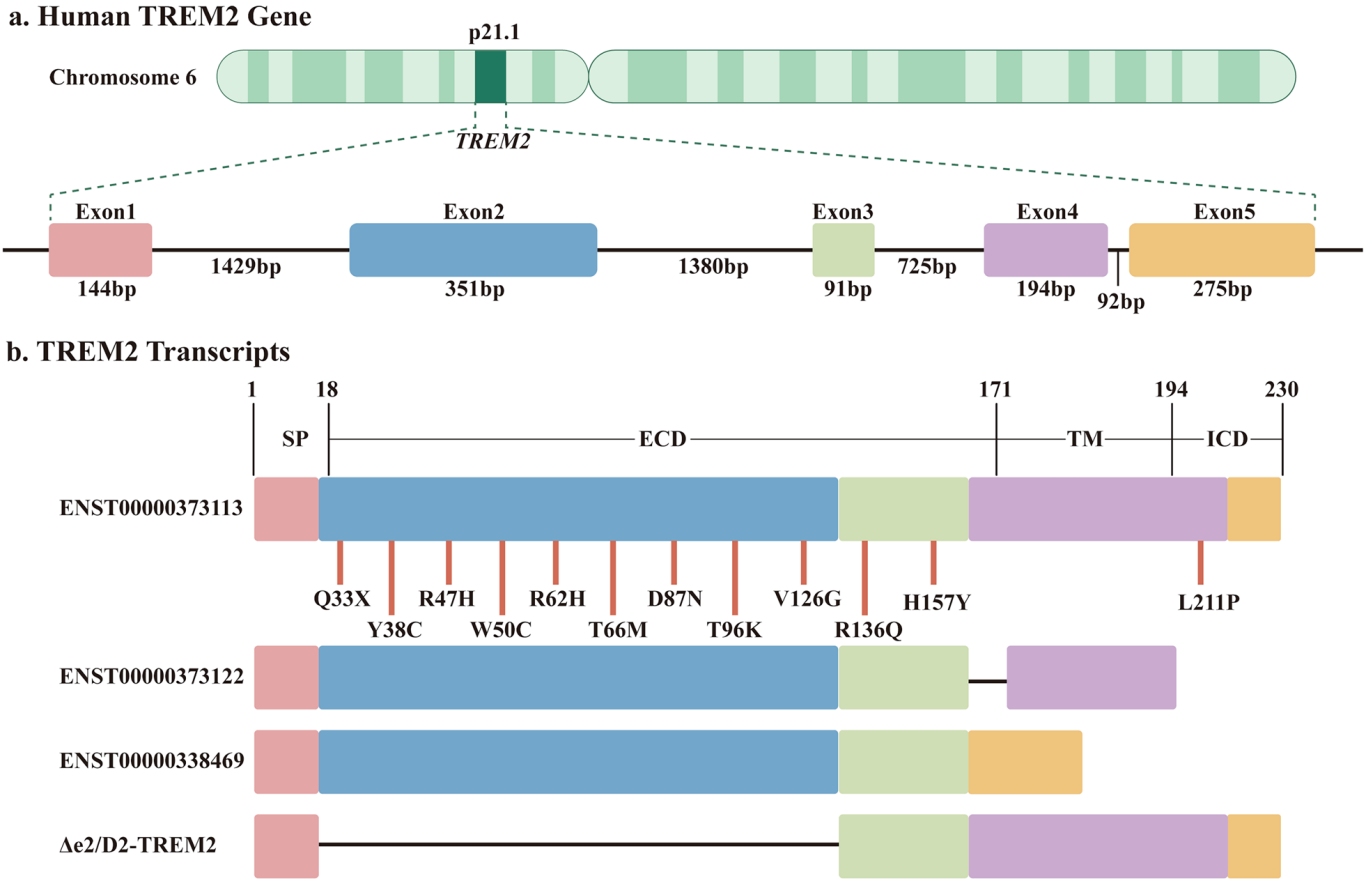
Schematic diagram of the *TREM2* gene and its transcripts. **a** The *TREM2* gene, located on chromosome 6p21.1, comprises five exons and undergoes transcription to generate pre-mRNA, which is subsequently spliced into mature transcripts. **b** The *TREM2* gene undergoes alternative splicing, producing four distinct transcripts. The canonical TREM2 protein, encoded by the full-length transcript, contains four main regions: the signal peptide (SP), extracellular domain (ECD), transmembrane region (TM) and intracellular domain (ICD). In **b**, Δe2/D2-TREM2 denotes the TREM2 isoform that is devoid of exon 2. Genetic variants of the *TREM2* gene result in diverse changes in the protein structure (shown above). TREM2, triggering receptor expressed on myeloid cells 2

The human *TREM2* gene is located on chromosome 6p21.1, while its murine ortholog resides on chromosome 17 (region C) (Fig. 1a) [12]. *TREM2* expression is strictly regulated by transcription factor YY1, which binds specifically to its promoter region [13, 14]. Additionally, *TREM2* transcription is modulated by DNA methylation [15–17]. In the human brain, four *TREM2* transcript variants have been identified, including ENST00000373113 (canonical), ENST00000338469 (lacking exon 4), ENST00000373122 (missing exon 5 and having an alternative start site at exon 4), and TREM2Δe2 (lacking exon 2) (Fig. 1b) [18–21]. The predominant isoform ENST00000373113 includes exon 5 and encodes a 230-amino acid transmembrane receptor protein [18, 19]. In contrast, the ENST00000373122 isoform lacks exon 5, resulting in a 222-amino acid transmembrane protein [18, 19]. Notably, ENST00000338469 lacks exon 4, resulting in a 219-amino acid secreted protein without a transmembrane domain [18, 19]. The TREM2Δe2 variant with exon 2 deletion produces a truncated 113-amino acid transmembrane protein that exhibits impaired ligand-binding capacity. This impairment stems from a partial loss of the signal peptide and the complete absence of the V-set immunoglobulin domain [20, 21]. Genetic variants such as R62H and T96K are associated with increased rates of exon 2 skipping. This enhanced skipping leads to reduced expression of full-length TREM2 and elevated levels of isoforms lacking exon 2 [22, 23].

The subcellular localization and dynamic distribution of TREM2 in microglia are precisely regulated by multiple signaling pathways. Synthesized and stored in the Golgi apparatus/trans-Golgi network, TREM2 undergoes dynamic trafficking to the plasma membrane upon cellular stimulation [24]. TREM2 internalization and recycling is regulated by multiple factors. The vacuolar protein sorting-associated protein 35 maintains the homeostatic distribution and functional integrity of TREM2 by mediating the endosomal recycling pathways [25, 26]. Presenilin 1 regulates the level of cell surface TREM2 and the phagocytic uptake of Aβ by microglia [27]. Conversely, the MEK1/2 signaling cascade functions as a negative regulator that suppresses TREM2 recruitment to the cell surface [28]. Notably, deficiency in neuraminidase 1 (NEU1), which normally catalyzes the removal of sialic acid residues from TREM2, leads to accumulation of sialylated TREM2 within the endo-lysosomal compartments and at the plasma membrane [29].

### TREM2 structure, ligand and signal transmission

TREM2 is a glycoprotein with a molecular weight of approximately 35–40 kDa, consisting of 230 amino acids [30]. Structurally, the protein is partitioned into four distinct functional regions: a signal peptide sequence, an extracellular domain, a transmembrane domain, and a cytoplasmic tail [31, 32]. Following ectodomain shedding, the membrane-bound C-terminal fragment undergoes γ-secretase-dependent intramembrane proteolysis, leading to its degradation and the release of the intracellular domain [33–35]. The extracellular domain adopts a stable V-shaped conformation, which is maintained by two pairs of highly conserved disulfide bonds (C36 ↔ C110 and C51 ↔ C60) within its single immunoglobulin-like fold [36, 37]. This domain contains two critical *N*-glycosylation sites at position N20 (immediately following the signal peptidase cleavage site) and N79, respectively, within the immunoglobulin-like V domain [38, 39]. Notably, glycosylation at N79 is crucial for stabilizing the protein structure and directly facilitates TREM2 trafficking to the cell surface. Moreover, glycosylation at both N20 and N79 sites is essential for efficient signal transduction [38, 39].

TREM2 exerts its biological functions by interacting with diverse ligands (Table 1). These include bacterial components [40, 41], heat shock protein 60 [42], apolipoprotein E (ApoE) [43, 44], low density lipoprotein and high density lipoprotein [45, 46], Aβ [47], phosphatidylserine (PS) [48–50], galectin-3 [51], cyclophilin A [52], TAR DNA-binding protein 43 (TDP-43) [53], complement component 1q (C1q) [54], interleukin (IL)-4 [55, 56] and IL-34 [57, 58]. The binding of these ligands to TREM2 exhibits complex competitive dynamics [59–61]. The hydrophobic site of TREM2 constitutes a pivotal region for ligand interactions, particularly for ApoE4 and TDP-43, while its basic site mainly mediates binding with C1q and IL-34 [59]. Within the hydrophobic site, ApoE4 and oligomeric Aβ_42_ (oAβ42) compete for binding, resulting in marked mutual inhibition of their binding capacities [59]. C1q also participates in the competitive interactions with both ApoE4 and oAβ_42_, though its impact on ApoE4 binding is comparatively modest, whereas ApoE4 and oAβ_42_ exhibit pronounced capacity to displace C1q from this site [59]. IL-34 and C1q share a partial binding interface on the basic site of TREM2, while the binding sites of IL-34 and ApoE4/oAβ_42_ on TREM2 are relatively independent, with minimal competitive binding [59].

**Table 1:** TREM2 ligands in Alzheimer’s disease

Ligand	TREM2 binding sites	Effects
Bacterial components [40, 41]	Extracellular domain	Promotes phagocytosis of bacteria
Heat shock protein 60 [42]	Extracellular domain	Promotes microglial phagocytosis
ApoE [43, 44]	Extracellular domain (hydrophobic site)	Promotes microglial uptake of Aβ
		Enhances phagocytosis of ApoE-bound apoptotic neurons
Aβ [47]	Extracellular domain	Promotes microglial phagocytosis
PS [48–50]	Extracellular domain	Enhances clearance of apoptotic cells
		Modulates lipid metabolism sensing
		Maintains neural homeostasis
Galectin-3 [51]	Extracellular domain	Exacerbates inflammatory response
		Exacerbates Aβ deposition
Cyclophilin A [52]	Extracellular domain (particularly the Pro144 residue)	Suppresses inflammatory response
TDP-43 [53]	Extracellular domain (hydrophobic site)	Enhances neuroprotective effects against TDP-43-related neurodegeneration
C1q [54]	Extracellular domain (particularly residues 31–71)	Suppresses the classical complement cascade
		Reduces complement-mediated synaptic loss
IL-4 [55, 56]	Extracellular domain (particularly residues 1–132)	Promotes anti-inflammatory response
IL-34 [57, 58]	Extracellular domain (particularly residues 1–132)	Suppresses inflammatory response
		Promotes neuronal and synaptic protection

TREM2, triggering receptor expressed on myeloid cells 2; ApoE, apolipoprotein E; Aβ, amyloid-β; PS, phosphatidylserine; TDP-43, TAR DNA-binding protein 43; C1q, Complement component 1q; IL-4, interleukin-4; IL-34, interleukin-34

Upon TREM2 binding to ligands, intracellular signaling pathways are activated through TREM2 adapter proteins DNAX-activation protein 12 (DAP12) and DAP10 [62]. DAP12 recruits and activates spleen tyrosine kinase (SYK), while DAP10 recruits and activates phosphatidylinositol 3-kinase (PI3K), initiating downstream signaling cascades [63]. The TREM2-DAP12 complex is critical for effective signal transduction [64]. At the cellular level, prior to assembly with TREM2, DAP12 is retained within the secretory pathway through interactions with the endoplasmic reticulum sorting receptor Rer1 [65]. Molecular analysis revealed that the hydrogen bonding between lysine 26 of TREM2 and aspartate 16 of DAP12 governs the formation of this stable complex [66].

Multiple mechanisms converge to negatively regulate TREM2 signaling. Leukocyte immunoglobulin-like receptor subfamily B member 2 (LILRB2) is an inhibitory receptor containing immunoreceptor tyrosine-based inhibitory motifs (ITIMs). LILRB2 inhibits TREM2 signaling in an ITIM-dependent manner, through LILRB2-TREM2 co-ligation by shared ligands (e.g., oAβ and PS) [67]. SH2-containing SHIP1 (inositol 5’-phosphatase 1) binds specifically to the phosphorylated ITAM (immunoreceptor tyrosine-based activation motif) of DAP12 through its SH2 domain. This binding prevents SYK recruitment and activation, thereby suppressing the downstream TREM2 signaling [68]. Additionally, NEU1 deficiency maintains TREM2 in a sialylated state. While the sialylated form of TREM2 can still associate with DAP12, SYK activation is severely impaired, ultimately attenuating TREM2 signaling [29].

### Generation of sTREM2

**Fig. 2:**
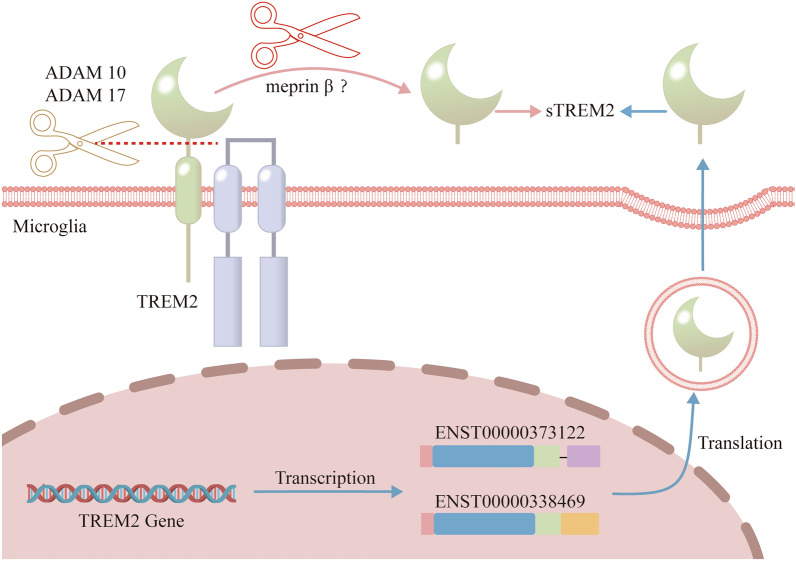
Generation of sTREM2. Red line: The extracellular domain of membrane-bound TREM2 is cleaved by α-secretase (e.g., ADAM10 and ADAM12) to produce sTREM2, and meprin β may also be involved in this process. Blue line: The transcripts ENST0000373122 and ENST0000338469, produced by transcription of the *TREM2* gene, undergo alternative splicing and are translated before being secreted extracellularly to form sTREM2. sTREM2, soluble triggering receptor expressed on myeloid cells 2; TREM2, triggering receptor expressed on myeloid cells 2; ADAM10, a disintegrin and metalloproteinase 10; ADAM12, a disintegrin and metalloproteinase 12

sTREM2 is a soluble protein with a molecular weight of ~ 20 kDa, consisting of 161 amino acids. sTREM2 is mainly present in the cerebrospinal fluid (CSF) and plasma [69]. sTREM2 is generated through two distinct mechanisms: proteolytic cleavage and alternative splicing (Fig. 2). In the proteolytic cleavage pathway, the cell surface TREM2 is cleaved by α-secretases (predominantly ADAM10 and ADAM17) between histidine at position 157 and serine at position 158. This results in the shedding of the ectodomain to form sTREM2 [70–73]. This process is positively regulated by the inactive rhomboid protein 2, which forms a regulatory complex with ADAM17, facilitating its maturation and activity [74]. In the alternative splicing pathway, specific transcripts (ENST00000373122 and ENST00000338469) undergo selective splicing and the encoded proteins are subsequently secreted into the extracellular space as sTREM2 [75].

The metalloproteinase meprin β promotes the shedding and degradation of TREM2 on the cell surface, but this effect may differ between macrophages and microglia [76]. Meprin β significantly induces sTREM2 release and degradation in macrophages, but its role in microglia has not been fully verified experimentally. Meprin β mediates TREM2 cleavage between arginine 136 and aspartic acid 137. Structural differences exist between the sTREM2 produced by ADAM10 and the sTREM2 sheared by meprin β [76]. Additionally, vascular endothelial growth factor enhances the activity of ADAM10/17, thereby promoting the cleavage of TREM2 [77]. Concurrently, interactions between BRI2 protein and TREM2 significantly inhibit the α-secretase-mediated processing, collectively regulating the generation of sTREM2 [78]. Any factor affecting BRI2 and vascular endothelial growth factor expression or function may indirectly influence sTREM2 levels. Therefore, future studies must consider these influences when assessing the accuracy of methods for detecting sTREM2 levels.

## TREM2 in AD pathology

Multiple studies in humans have demonstrated significantly elevated levels of TREM2 in the plasma of patients with AD and mild cognitive impairment (MCI) compared to cognitively unimpaired controls [79–82]. Among the MCI patients, those who later progressed to AD exhibited significantly higher peripheral blood *TREM2* mRNA expression than non-converters [81, 83]. Histopathological analyses revealed a significant association between elevated TREM2 expression and the hallmark features of AD: Aβ deposition, tau protein phosphorylation, and presynaptic protein loss [84–87]. Notably, individuals exhibiting significant AD-related neuropathological burden but maintaining cognitively unimpaired status often display higher TREM2 levels than typical AD patients [88]. This key observation supports the hypothesis that upregulation of TREM2 may represent an early compensatory, neuroprotective response during the preclinical or prodromal stages of AD. In the following, we will explore the molecular interactions between TREM2 and AD pathogenesis.

### TREM2 mediates microglial migration and metabolism

TREM2 serves as a receptor on the microglial cell surface and regulates microglial functions such as phagocytosis, chemotaxis, and migration. It precisely modulates the directional migration capacity of microglia through the purinergic receptor-mediated calcium signaling [8]. TREM2 deficiency or dysfunction not only significantly impairs the overall migratory ability of microglia, but also causes directional migration defects, thereby reducing the response efficiency of microglia to pathological processes such as neuronal damage [8, 89].

TREM2 also plays a crucial role in glucose and lipid metabolism. TREM2 maintains the normal metabolic activities of microglia by promoting oxidative phosphorylation [5, 90]. TREM2 deficiency leads to a decline of function and metabolic efficiency of glucose transporters in microglia, resulting in reduced cerebral glucose uptake in animal models, as evidenced by decreased signal intensity in [^18^F]fluorodeoxyglucose positron emission tomography (FDG-PET) imaging [91]. In lipid metabolism, TREM2 maintains brain cholesterol homeostasis by regulating cholesterol metabolism in microglia, thereby preventing pathological lipid accumulation [7]. Additionally, the TREM2-dependent process of lipid droplet biogenesis in phagocytes is equally indispensable for remyelination [92]. In demyelination injury models, TREM2-deficient mice exhibit significant reduction in lipid droplet formation, leading to accumulation of free cholesterol that triggers endoplasmic reticulum stress, ultimately exacerbating defects in myelin repair [92]. TREM2 dysfunction in lipid metabolism is also involved in many other diseases [93]. It is important to note that TREM2 can influence neuronal mitochondrial function and ATP production indirectly, by regulating the metabolically supportive functions of microglia (such as clearing metabolic waste) and their immunometabolic state. This ensures energy supply for synaptic transmission and neural activity [9].

### TREM2 maintains the microglial inflammatory response equilibrium

In AD, pathological proteins such as Aβ and phosphorylated tau can activate microglia and trigger microglial secretion of proinflammatory mediators that accelerate disease progression. Increased TREM2 expression effectively suppresses neuroinflammation, whereas TREM2 dysfunction amplifies the inflammatory response [94, 95]. At the cellular level, TREM2 facilitates microglial transformation from the pro-inflammatory to the immunoregulatory phenotype, the latter characterized by reduced production of proinflammatory cytokines coupled with increased secretion of anti-inflammatory cytokines and brain-derived neurotrophic factor [96–98]. This phenotypic shift not only attenuates the neuroinflammatory cascade, but also establishes a microenvironment conducive to neuronal repair and synaptic remodeling [98]. Through integrated analyses of genomic profiles, morphological traits, and functional specializations, researchers have characterized distinct microglial subtypes associated with TREM2 functionality in AD pathogenesis (Table 2) [99–106]. Particular emphasis is placed on the pivotal role of TREM2 in the second stage of disease-associated microglia (DAM) activation [100]. During this stage, TREM2 acts as a central regulator by upregulating genes critical for lipid metabolism and phagocytic activity [100]. In addition, TREM2 deficiency disrupts the full activation of DAM, leading to profound deficits in phagocytic capacity and lipid metabolic efficiency [100].

**Table 2:** Involvement of TREM2 in the heterogeneity of microglial phenotypes

Cell phenotype	Examples of gene expression		Effects of TREM2	Primary functions
	Upregulated genes	Downregulated genes		
Disease-associated microglia (DAM) [100]	*Apoe, Ctsd, Lpl, Tyrobp, Trem2*	*Cx3cr1, P2ry12, Tmem119*	Involved in the second phase of DAM activation	Restricts neurodegeneration
				Involved in Aβ clearance
Neurodegenerative microglia (MGnD) [101, 104]	*Apoe, Spp1, Itgax, Axl, Lilrb4, Clec7a, Csf1*	*P2ry12, Tmem119, Gpr34, Csf1r, Hexb, Rhob, Cx3cr1*	Triggers ApoE signaling, driving MGnD transcriptional phenotype	Regulates neuronal survival Induces chronic inflammation Enhances tau propagation
Amyloid‑responsive microglia (ARM) [102, 103]	*Apoe, H2-ab1, H2-eb1, Cst7, Spp1, Gpnmb, Dkk2*	*Siglech, Inpp5d, Bin1, Ms4a6b, Ctsf*	Maintains the ARM response to Aβ	Involved in Aβ clearance
Dark microglia (DM) [99, 105]	*Atf4, Fasn*	*Ppp1r15a, Asns, Wfs1, Vldlr, Aldh18a1, Fasn*	Maintains the abundance and functional role of DM	Increases Aβ plaques
				Exacerbates tau spread
				Pruns synapses excessively
				Excretes toxic lipids
Senescent microglia [106]	*Trem2, Apoe, Cd9, Cd11c*	Not reported	Senescent microglia express high levels of TREM2	Exacerbates chronic inflammation
				Overexpression of senescence-related proteins p16, p19, p21, γH2AX, and p53
White matter-associated microglia [360]	*ApoE, Cst7, Bm2, Lyz2, Cd63, Clec7a, Ctsb, Ctss, Ctsz, H2-D1, H2-K1*	*P2ry12, P2ry13, Csfr1r, Cx3cr1, Hexb, Tmem119*	Promotes the formation and function of white matter-associated microglia	Involved in the clearance of degenerating myelin debris

TREM2, triggering receptor expressed on myeloid cells 2; AD, Alzheimer’s disease; APOE, Apolipoprotein E; *Ctsd*, Cathepsin D; *Lpl*, lipoprotein lipase; *Tyrobp*, TYRO protein tyrosine kinase binding protein; *Cx3cr1*, C-X3-C motif chemokine receptor 1; *P2ry12*, purinergic receptor P2Y12; *Tmem119*, transmembrane protein 119; Aβ, amyloid-β;*SPP1*, secreted phosphoprotein 1; *Itgax*, Integrin alpha X; *Axl*, Anexelekto; *Lilrb4*, leukocyte immunoglobulin-like receptor B4; *Clec7a*, C-type lectin domain family 7A; *Csf1*, Colony stimulating factor 1; *Gpr34*, G protein-coupled receptor 34; *Csf1r*, Colony stimulating factor 1 receptor; *Hexb*, Hexosaminidase B; *Rhob*, Ras homolog family member B; *H2-ab1*, Histocompatibility 2, class II antigen A, beta 1; *H2-eb1*, Histocompatibility 2, class II antigen E, beta 1; *Cst7*, Cystatin F; *Gpnmb*, Glycoprotein non-metastatic melanoma protein b; *Dkk2*, Dickkopf-related protein 2; *Siglech*, Sialic acid-binding immunoglobulin-like lectins H; *Inpp5d*, Inositol polyphosphate-5-phosphatase D; *Bin1*, Bridging integrator 1; *Ms4a6b*, Membrane spanning 4-domain, subfamily A, member 6B; *Ctsf*, Cathepsin F; *Atf4*, activating transcription factor 4; *Fasn*, Fatty acid synthase; *Ppp1r15a*, protein phosphatase 1 regulatory subunit 15A; *Asns*, asparagine synthetase; *Wfs1*, Wolfram syndrome 1; *Vldlr*, very low density lipoprotein receptor; *Aldh18a1*, Aldehyde dehydrogenase 18 family member A1; *Cd9*, CD9 molecule; *Cd11c*, Complement receptor 4; *Bm2*, Bactericidal/permeability-increasing protein 2; *Lyz2*, Lysozyme 2; *Cd63*, Cluster of differentiation 63; *Ctsb*, Cathepsin B; *Ctss*, Cathepsin s; *Ctsz*, Cathepsin z; *H2-D1*, histocompatibility 2, D region locus 1; *H2-K1*, histocompatibility 2, K1, K region; *P2ry13*, purinergic receptor P2Y13; *Csfr1r*, Colony stimulating factor 1 receptor

**Fig. 3:**
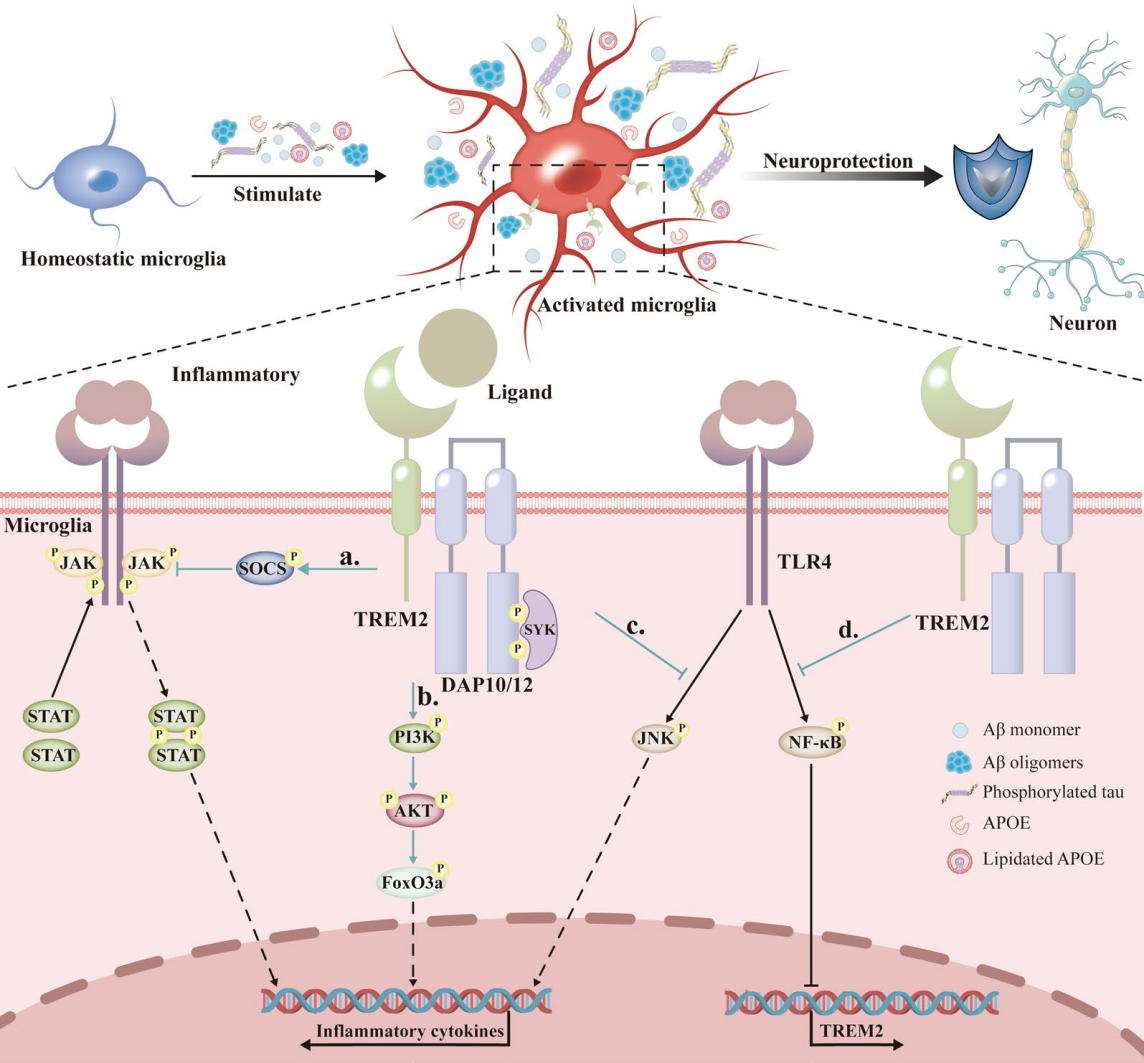
The TREM2-mediated signaling pathways in microglia modulate AD-related inflammation. At the onset of inflammation, TREM2 on microglia senses pathological proteins in AD, such as Aβ monomer, Aβ oligomers, phosphorylated tau protein, ApoE, and lipidated ApoE. This triggers their transition from a homeostatic state to an activated state, exerting neuroprotective effects. **a** Specifically, inflammatory stimuli engage corresponding receptors on microglial cells, triggering the activation of the JAK/STAT signaling pathway. TREM2 promotes the phosphorylation of SOCS, which in turn inhibits STAT phosphorylation, thereby alleviating inflammation. **b** TREM2 activates the PI3K/AKT signaling pathway, leading to AKT-mediated phosphorylation of FoxO3a. This phosphorylation event induces FoxO3a translocation from the nucleus to the cytoplasm, reducing its nuclear levels and suppressing the expression of pro-inflammatory genes. **c** Inflammatory stimuli trigger TLR4 receptors, subsequently driving the inflammatory process via the JNK signaling pathway. Conversely, TREM2 exerts an inhibitory effect on JNK phosphorylation, thereby curbing the inflammatory response. **d** The TLR4 receptor inhibits TREM2 expression by promoting NF-κB phosphorylation, whereas TREM2 reciprocally suppresses NF-κB phosphorylation through PI3K/AKT signaling. TREM2, triggering receptor expressed on myeloid cells 2; AD, Alzheimer’s disease; Aβ, amyloid-β; ApoE, apolipoprotein E; JAK, Janus kinase; STAT, signal transducer and activator of transcription; SOCS, suppressor of cytokine signaling; PI3K, phosphoinositide 3-kinase; AKT, Protein Kinase B; FoxO3a, Forkhead box O3a; TLR4, Toll-like receptor 4; JNK, c-Jun N-terminal kinase; NF-κB, nuclear factor kappa-B

From the signal transduction perspective, TREM2 modulates inflammation through multiple pathways including the JNK, PI3K/AKT/FoxO3a and JAK/STAT/SOCS pathways (Fig. 3) [107–112]. For instance, Toll-like receptor 4 (TLR4) activation triggers release of inflammatory factors and exacerbates neuroinflammation, while TREM2 negatively regulates the TLR4 signaling, thereby reducing inflammation [55, 113–116]. Molecules such as phospholipase Cγ2 and paraoxonase 1 also participate in the TREM2-mediated inflammatory regulation [117, 118]. Studies on TREM2-mediated exosome release have revealed stimulus-dependent proteomic differences: lipopolysaccharide (LPS) stimulation enhances inflammatory exosome release, whereas phosphatidylserine promotes metabolic exosome secretion [119].

Inflammation can affect TREM2 expression, and vice versa. On the one hand, inflammatory stimuli significantly inhibit microglial TREM2 expression, compromising the anti-inflammatory capacity and disrupting the microglial equilibrium (Fig. 3) [107, 120, 121]. On the other hand, anti-inflammatory cytokines like IL-4 and IL-10 upregulate microglial TREM2 [122]. The combination of IL-34 with TREM2 effectively inhibits NLRP3 inflammasome activation and release of proinflammatory cytokines, thereby mitigating neuroinflammation [123].

### TREM2 interactions with proteins

#### Aβ

**Fig. 4:**
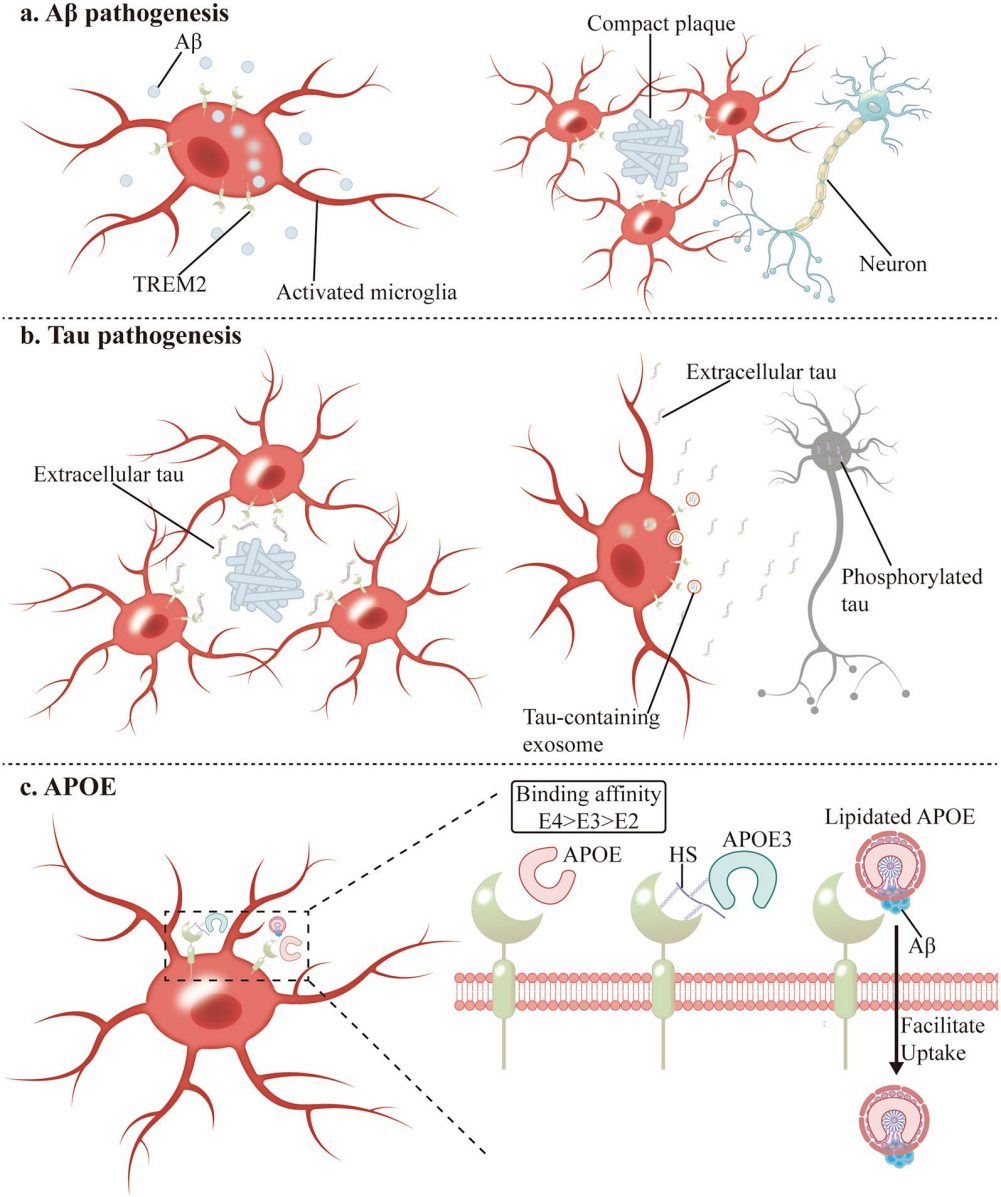
TREM2 interacts with proteins. **a** In the Aβ seeding stage, activated microglia capture Aβ seeds via TREM2-Aβ interaction, facilitating their clearance. As plaques accumulate, microglia cluster around them in a TREM2-dependent manner and prune Aβ fibrils at plaque edges, thereby compacting the plaques. Plaque-associated microglia also form a protective barrier, mitigating Aβ neurotoxicity to surrounding nerve fibers. **b** When Aβ-related tau pathology arises, TREM2-dependent microglia cluster around tau aggregates, thereby restricting their seeding and propagation. Additionally, TREM2 directly clears extracellular phosphorylated tau by engulfing tau-laden extracellular vesicles. **c** The binding affinity of ApoE isoforms to TREM2 follows the order E4 > E3 > E2. When ApoE3 binds to TREM2, HS acts as a bridging molecule. Lipidated ApoE enhances Aβ uptake by TREM2 via direct interaction with Aβ aggregates. TREM2, triggering receptor expressed on myeloid cells 2; Aβ, amyloid-β; ApoE, apolipoprotein E; HS, heparan sulfate

TREM2 is a high-affinity receptor for Aβ, exhibiting significantly greater binding affinity for Aβ oligomers compared to monomers [47, 60, 61, 124, 125]. Functional TREM2 signaling is essential for directing microglial responses to Aβ deposits, facilitating their recruitment and accumulation around amyloid plaques (Fig. 4a) [124, 126–132]. In the second stage of DAM activation, TREM2 mediates the migration of microglia toward Aβ plaques and their dense clustering around them, which enhances local phagocytosis to clear Aβ [100]. Upregulation of TREM2 expression enhances the phagocytic function of microglia toward Aβ, accelerates the clearance of Aβ, and simultaneously alleviates Aβ-associated neuroinflammation [133–136]. TREM2 mediates microglial phagocytosis of Aβ through multiple signaling pathways. For example, TREM2 upregulates the C/EBP-α-dependent CD36 expression in microglia [137], and promotes Aβ phagocytosis through SYK-dependent and -independent pathways [138]. Exosomes are also involved [139]. Recent studies have further revealed that TREM2 mediates the migration of microglia to amyloid plaques surrounded by phosphatidylserine and promotes microglial phagocytosis and clearance of these plaques [140].

TREM2 significantly mitigates the propagation and toxicity of Aβ plaques by preserving the microglial barrier function. Microglia with high TREM2 expression tightly wrap early Aβ plaques, thus promoting their compaction and isolation [141, 142]. Conversely, deficiencies in TREM2 function significantly impair the ability of microglia to form an Aβ barrier, resulting in reduced compaction of Aβ plaques and aggravation of axon malnutrition [143]. TREM2 functional defects lead to differential accumulation of modified and unmodified Aβ species in extracellular plaques and intraneuronal deposits, thereby affecting the morphological characteristics and toxicity of Aβ plaques [144]. In addition, PET imaging has revealed the effect of TREM2 functional status on the proportion of fibrous and non-fibrous components in Aβ plaques. TREM2 knockout (KO) mice show a higher proportion of non-fibrous Aβ components, which impacts the Aβ PET signal [145].

#### Tau

TREM2 plays a crucial role in limiting the spread of tau pathology. In bioinformatics sequencing studies of animal models with tau pathology, changes in *TREM2* gene expression are significantly correlated with the progression of tau pathology [146–148]. Enhancing TREM2 expression in tauopathy models with intact TREM2 function can attenuate tau pathology and neurodegenerative phenotypes [149]. Mechanistically, TREM2 suppresses tau hyperphosphorylation and neuronal apoptosis through activation of the PI3K/AKT/GSK-3β signaling cascade [150]. At the cellular level, TREM2 modulates microglial inflammatory responses to decelerate tauopathy progression [151]. Compared with wild-type mice, *TREM2* KO mice exhibit altered tau metabolic profiles toward extracellular vesicle release and advanced aggregation states rather than lysosomal degradation pathways (Fig. 4b) [152].

However, discrepancies exist in the reported effects of *TREM2* KO on tau pathology progression across studies. In Aβ-containing models, TREM2 deficiency exacerbates tau pathology, whereas in pure tauopathy mouse models, it may attenuate tau propagation [153–158]. Specifically, under Aβ pathological conditions, TREM2 deficiency significantly promotes tau pathology progression and brain atrophy, potentially because TREM2 is involved in microglial transition from homeostatic to DAM and thus is protective in the context of Aβ pathology (Fig. 4b) [155–157]. On the contrary, in pure tauopathy mouse models, TREM2 KO mice exhibit markedly reduced tau pathology, accompanied by decreased microglial density and downregulation of neuroinflammatory genes, suggesting that TREM2 deficiency may mitigate tau propagation by suppressing microglial activation or inflammatory signaling pathways [158]. Therefore, the role of TREM2 in tau pathology appears to be influenced by multiple factors, including the presence of Aβ pathology, the stage of tau pathology, and the model used [153, 154, 158]. Future research is needed to elucidate the stage- and model-specific involvement of TREM2 in tau pathology progression, with a particular focus on its impacts on microglial function, neuroinflammation, and tau propagation across pathological stages and models.

#### ApoE

Epidemiological studies and GWAS have established *APOE4* as the strongest single genetic risk factor for late-onset AD [159, 160]. The human *APOE* gene exhibits three alleles (ε2, ε3, ε4) encoding three protein isoforms: ApoE2, ApoE3, and ApoE4 [161]. In terms of AD risk, ApoE3 is considered neutral, ApoE2 demonstrates protective effects, while ApoE4 exerts deleterious impacts [161]. The presence of *APOE* ε4 allele increases the AD risk and lowers the onset age in a dose-dependent manner, underscoring its pivotal role in AD pathogenesis [161].

Multiple omics studies have demonstrated an interaction between TREM2 and ApoE during the progression of AD [162–164]. At the molecular level, ApoE functions as a high-affinity ligand for TREM2 [43–45], with its hinge region being critical for TREM2 binding [165]. The binding affinities differ significantly among ApoE subtypes: ApoE4 > ApoE3 > ApoE2 (Fig. 4c) [60, 61, 165, 166]. Notably, lipidation of ApoE enhances its TREM2 binding capacity [45]. Interestingly, heparan sulfate acts as a co-receptor in the TREM2-ApoE3 interaction [167]. Upon 6-O-sulfation, heparan sulfate binds microglial TREM2 while simultaneously associating with ApoE3, forming a ternary complex (Fig. 4c).

At the cellular level, Aβ-ApoE complexes are phagocytosed more efficiently via TREM2-mediated microglial pathways (Fig. 4c) [45]. In APP/PS1 transgenic mice, TREM2 KO reduced both ApoE deposition around plaques and microglial clustering compared to wild-type controls [168]. Activation of the TREM2-ApoE axis promotes microglial phenotypic transition from homeostatic to neurodegenerative states, impacting AD progression [101, 166].

TREM2 expression and function vary with ApoE subtype. Under inflammatory conditions, TREM2 levels in *APOE4* mice are lower than those in *APOE3* mice [169]. In *APOE3* mice, male mice exhibit higher TREM2 expression levels than female mice in microglia associated with amyloid plaques [170]. In vitro studies demonstrated that TREM2 levels were significantly elevated in the ApoE4-treated group compared to the ApoE3-treated group [171]. Mechanistically, ApoE4 activates SYK more efficiently than ApoE2 through TREM2-dependent mechanisms, suggesting differential signal transduction effects [172].

In *TREM2* KO mice, ApoE3 and ApoE4 differentially affect AD pathology. *APOE3* carriers show milder cognitive deficits than *APOE4* carriers [173], with preserved microglial Aβ clearance capacity even in the absence of TREM2 [174]. Conversely, *APOE4* knock-in mice with *TREM2* KO exhibit accelerated tau pathology, including enhanced aggregation and neurodegeneration, compared to wild-type mice  [175]. These findings underscore the need for further investigation into the TREM2-ApoE interactions in AD pathogenesis, particularly their roles in microglial function and tauopathy modulation.

### TREM2 maintains synaptic homeostasis

**Fig. 5:**
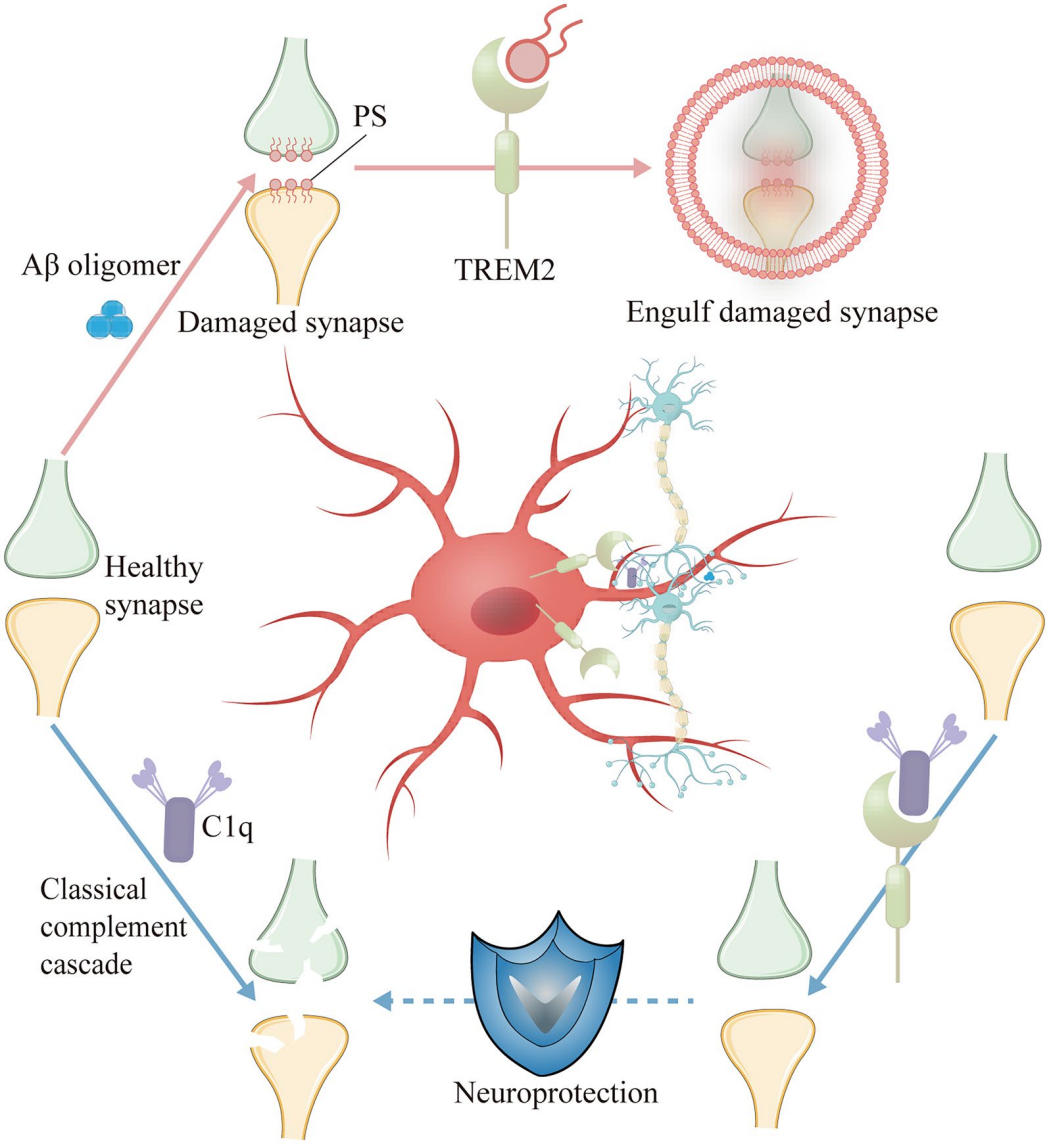
TREM2 maintains synaptic homeostasis through dual neuroprotective mechanisms. Red Line: When stimulated by Aβ oligomers, synapses exhibit hyperactivity and phosphatidylserine externalization. TREM2 recognizes exposed phosphatidylserine, triggering microglial phagocytosis of damaged synapses to promote neuronal homeostasis. Blue Line: During neurodegeneration, TREM2 competitively binds to C1q, inhibiting classical complement pathway activation and preventing complement-mediated synaptic loss. TREM2, triggering receptor expressed on myeloid cells 2; Aβ, amyloid-β; PS, phosphatidylserine; C1q, complement component 1q

TREM2 preserves synaptic homeostasis through two interdependent mechanisms: phagocytic clearance of dysfunctional synapses to maintain neural network precision, and establishment of a negative feedback loop with the complement system that suppresses aberrant activation. Therefore, TREM2 provides dual protection for synaptic integrity [176, 177]. Recent evidence shows that at postsynaptic spines, Aβ oligomer stimulation induces phosphatidylserine externalization to the outer membrane, generating a TREM2-specific phagocytosis signal (Fig. 5) [50]. This targeted engulfment of tagged synapses alleviates Aβ-associated synaptic hyperactivity [178], thereby promoting restoration of both synaptic plasticity and network functionality while mitigating pathological markers. Critically, the TREM2 R47H variant impairs the microglia-mediated synaptic pruning [178, 179]. In mouse models, the R47H variant increases cortical synaptic density and exacerbates epileptiform activity, potentially due to the defects in the clearance of hyperactive synapses [178, 179]. These findings underscore the critical role of TREM2 in maintaining the neural circuit balance through synaptic pruning.

Concurrently, the high-affinity binding between TREM2 and C1q sterically occludes the complement cascade initiation sites (Fig. 5) [54], preventing synaptic vulnerability to overactivation. In AD models, TREM2 haploinsufficiency increased the complement-mediated synaptic loss, underscoring the neuroprotective role of TREM2. Administration of a 41-amino-acid TREM2 peptide recapitulates this inhibitory effect by engaging C1q, thereby attenuating microglial phagocytosis and complement-mediated synaptic loss in advanced AD [54]. Collectively, TREM2 is a critical complement modulator with neuroprotective roles, and may be a novel therapeutic strategy for neurodegenerative disorders including AD.

## sTREM2 in AD

In human studies, sTREM2 levels in the CSF or plasma exhibit dynamic fluctuations during AD progression, supporting its potential as a biomarker for AD [180]. These fluctuations not only correlate with the expression profiles of various inflammatory mediators, but also specifically associate with advancing of Aβ deposition and tau pathology [181–183]. Such evidence collectively implicates sTREM2 as a critical mediator in AD pathogenesis. In the following, we will systematically discuss (1) the correlations between sTREM2 dynamics and AD-related neuropathological hallmarks; (2) the interplay between sTREM2 expression and AD-risk genetic modifiers; and (3) effects of sTREM2 on Aβ accumulation, tau hyperphosphorylation, and synaptic dysfunction.

### Correlation between sTREM2 and AD neuropathology

**Fig. 6:**
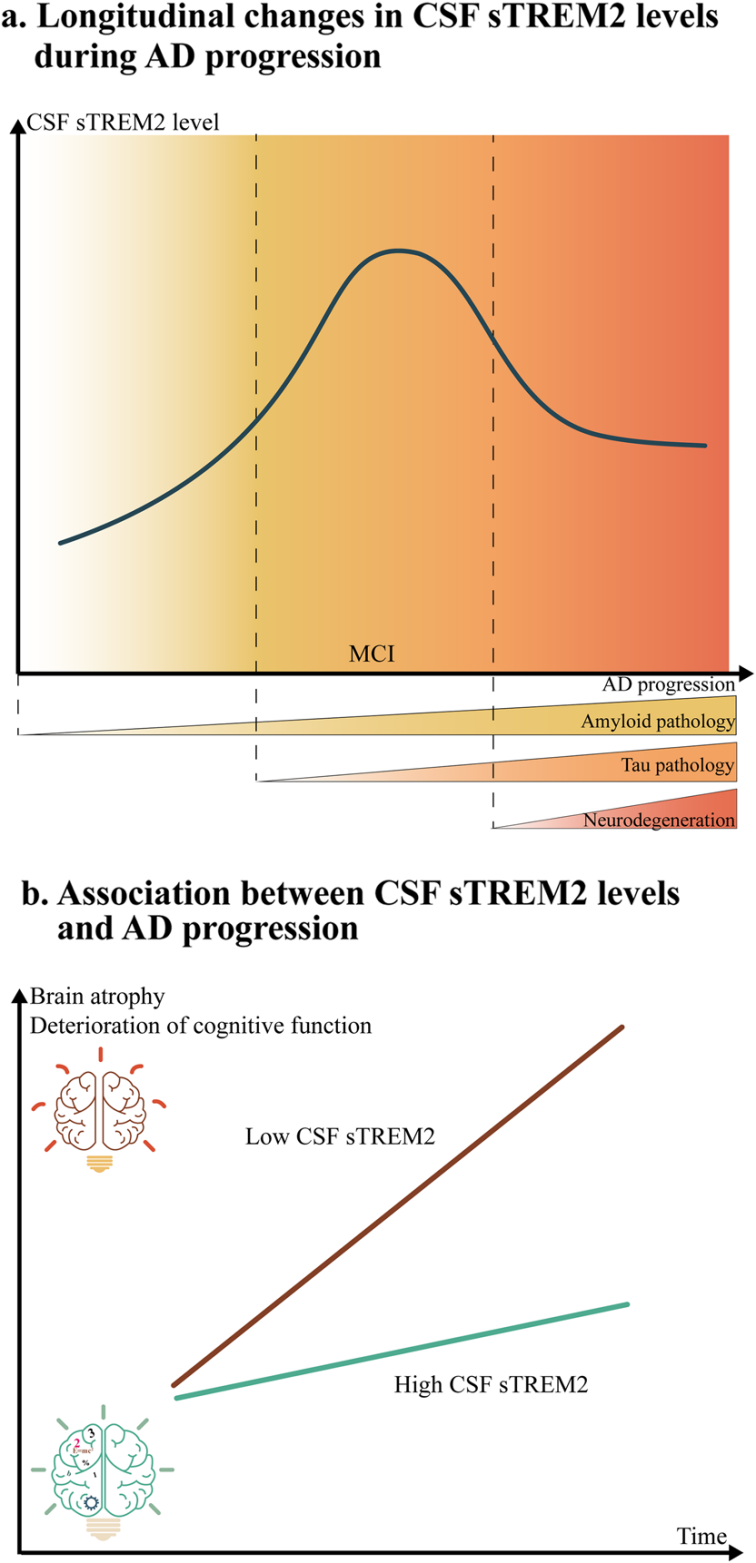
Clinical significance of CSF sTREM2 levels in the progression of AD. **a** Longitudinal changes in CSF sTREM2 levels during AD progression. **b** Association between CSF sTREM2 levels and AD progression. Individuals with high levels of CSF sTREM2 exhibit slower brain atrophy and cognitive decline compared to those with low levels of CSF sTREM2. CSF, cerebrospinal fluid; sTREM2, soluble triggering receptor expressed on myeloid cells 2; AD, Alzheimer’s disease; MCI, mild cognitive impairment

Human studies on AD consistently demonstrate dynamic fluctuations in CSF sTREM2 levels across disease progression, with peak concentrations specifically observed during the MCI phase (Fig. 6a) [184–198]. Studies in the United States, Sweden, the United Kingdom, and Poland all revealed that CSF sTREM2 levels in AD patients are significantly higher than those in cognitively normal controls, with MCI patients exhibiting highest concentrations [185, 187, 194, 196]. A Chinese study categorizing participants by AD pathological markers identified dynamic changes in CSF sTREM2 levels across four preclinical AD stages, closely tracking the neuropathological progression [189]. Concurrently, multiple studies have shown positive correlations between CSF sTREM2 and total tau protein/tau phosphorylation levels [184, 185, 187–191, 193, 196]. In AD patients with abnormal Aβ pathology, higher plasma sTREM2 levels are associated with reduced cerebral tau accumulation [197]. Notably, findings regarding the sTREM2–Aβ relationship varied significantly across studies [188, 191, 193], particularly for Aβ_1-42_ [184, 189]. The Memory and Aging Project found no correlation between CSF sTREM2 and Aβ_1-42_, but identified a significant positive association with shorter Aβ fragments (Aβ_x-40_ and Aβ_x-42_) [191]. These discordant results highlight the complexity of sTREM2–Aβ interactions, which require further elucidation.

Longitudinal studies [199–208] have revealed that elevated CSF sTREM2 levels are associated with slower clinical progression in Aβ-positive AD patients [200, 204]. This protective association correlates with reduced Aβ deposition and tau aggregation [200, 203, 206]. Additionally, individuals with higher baseline microglial activation markers exhibit accelerated CSF sTREM2 increases [207], suggesting potential links between microglial metabolic activity and sTREM2 dynamics [201, 205].

Based on the A/T/N classification system, dynamic changes in CSF sTREM2 levels are observed across stages of AD progression [209–215]. Cross-sectional analyses in three cohorts demonstrated that elevated CSF sTREM2 concentrations in AD patients correlate with tau-mediated neurodegeneration rather than reduced Aβ plaque burden in the A^+^T^−^ populations [209, 212, 214]. Paradoxically, during the preclinical phase of late-onset AD (A^+^T^−^N^−^), CSF sTREM2 levels decline [209]. This phenomenon may reflect microglial encapsulation of Aβ plaques in early pathogenesis, potentially trapping sTREM2 within plaques and limiting its diffusion into the CSF, a mechanism that requires further validation. Four longitudinal studies revealed an inverse relationship between increasing CSF sTREM2 and tau aggregation in A^+^T^+^ samples [210, 211, 213, 215]. Specifically, an elevated sTREM2/p-tau_181_ ratio in the CSF is associated with attenuated progression from MCI to dementia in A^+^T^+^ patients [211], suggesting potential neuroprotective roles of sTREM2 in tauopathy-dominant AD stages. Thus, higher CSF sTREM2 levels are associated with a slower rate of cognitive decline (Fig. 6b).

Consistently, neuroimaging studies in AD patients have revealed that higher CSF sTREM2 levels correlate with slower rates of brain atrophy (Fig. 6b) [203, 216–220]. A Barcelona-based cross-sectional study identified a positive correlation between CSF sTREM2 concentration and gray matter volumes in critical regions, such as the bilateral inferior/middle temporal cortices, precuneus, superior limbic gyri, and angular gyri, among patients with MCI [216]. Subsequent longitudinal investigations have further elucidated the stage-specific roles of sTREM2 during AD progression [203, 217–220]. Notably, MCI patients with higher baseline CSF sTREM2 exhibited a significantly slower gray matter atrophy rate in parahippocampal gyrus and left fusiform cortex [219]. In contrast, AD patients presenting higher sTREM2 levels showed faster mean diffusivity in the bilateral posterior corona radiata and right superior longitudinal fasciculus [219]. These divergent findings suggest a dual role for sTREM2: it exerts neuroprotective effects during early disease stages while this protective capacity is lost with progression of neurodegeneration. In autosomal-dominant AD carriers, CSF sTREM2 elevations are detectable as early as 21 years before symptom onset, with concentrations rising steadily toward clinical manifestation [203, 217]. Moreover, higher baseline sTREM2 levels correlate with attenuated hippocampal volume loss, particularly in individuals demonstrating progressive sTREM2 increases [203, 217]. Collectively, these data indicate that while elevated CSF sTREM2 is associated with neurodegenerative processes and gray matter atrophy, it may simultaneously confer regional neuroprotection during the preclinical stages of AD.

Multiple factors, such as ethnicity, subclinical psychological state (e.g., mild depressive symptoms), and genetic predisposition, may influence the level of CSF sTREM2 [208, 221, 222]. Consequently, these variables should be systematically controlled during data analysis and interpretation of results, in order to maintain the validity and reproducibility of research findings.

### Genetic modifiers of CSF sTREM2

Previous in-depth GWAS studies have identified a significant association between variations in the *MS4A* gene cluster on chromosome 11 and CSF sTREM2 levels. This association specifically involves single nucleotide polymorphisms rs7232, rs1582763, and rs6591561 [223–225]. The rs1582763 variant correlates with elevated CSF sTREM2 levels and reduced AD risk, whereas the rs6591561 variant is associated with decreased CSF sTREM2 levels and increased AD risk [224, 226]. Mechanistic investigations suggested that the protective variant rs1582763 attenuates the correlation between CSF sTREM2 and Aβ_40_ levels, while the risk variant rs6591561 enhances this relationship in certain cohort [227]. Notably, the rs1582763 minor allele A strengthens the association between CSF sTREM2 levels and blood–brain barrier dysfunction, implying a crosstalk between peripheral and central immune responses. Importantly, the *MS4A* gene cluster variations primarily influence Aβ levels and blood–brain barrier permeability rather than tau-related pathologies.

Apart from *MS4A* gene cluster variations, *TGFBR2* and *NECTIN2* are emerging as novel regulators of sTREM2 biological function [228]. Experimental overexpression of *TGFBR2* increases CSF sTREM2 levels, while knockdown of *TGFBR2* decreased these levels. Similarly, *NECTIN2* overexpression increases the CSF sTREM2 concentration. Despite limitations including sample size constraints and incomplete functional characterization of regulatory factors, these discoveries warrant further mechanistic investigations.

*TREM2* variants exhibit heterogeneous effects on CSF sTREM2 levels (Table 3). Pathogenic variants Q33X, T66M, D87N, T96K, R136Q and L211P reduce CSF sTREM2 levels compared to controls [209, 223]. Conversely, R47H- and H157Y-carriers show significantly elevated CSF sTREM2 levels, suggesting distinct AD risk mechanisms compared to other TREM2 variants [209]. In contrast, plasma sTREM2 analyses revealed no variant-associated differences [229], underscoring the complexity of *TREM2* variant effects and providing critical avenues for molecular mechanism studies.

**Table 3:** Summary of diseases related to *TREM2* variants and CSF sTREM2 levels

Variant	Related diseases	CSF sTREM2 level
Q33X	Nasu-Hakola disease [237], frontotemporal dementia [241]	Reduced [223]
Y38C	Frontotemporal dementia [241]	N/A
R47H	Alzheimer’s disease [10, 11]	Increased [209, 223]
W50C	Nasu-Hakola disease [238]	N/A
R62H	Alzheimer’s disease [18]	No change [223]
T66M	Frontotemporal dementia [241, 385]	Reduced [223]
D87N	Alzheimer’s disease [10]	Reduced [223]
T96K	Frontotemporal disease [386]	Reduced [223]
V126G	Nasu-Hakola disease [387]	N/A
R136Q	Alzheimers disease [274]	Reduced [223]
H157Y	Alzheimers disease [388]	Increased [209]
L211P	Alzheimers disease [389], frontotemporal disease [386]	Reduced [209, 223]

TREM2,triggering receptor expressed on myeloid cells 2; CSF, cerebrospinal fluid; sTREM2, soluble triggering receptor expressed on myeloid cells 2; N/A, not applicable

### Roles of sTREM2 in AD pathology

**Fig. 7:**
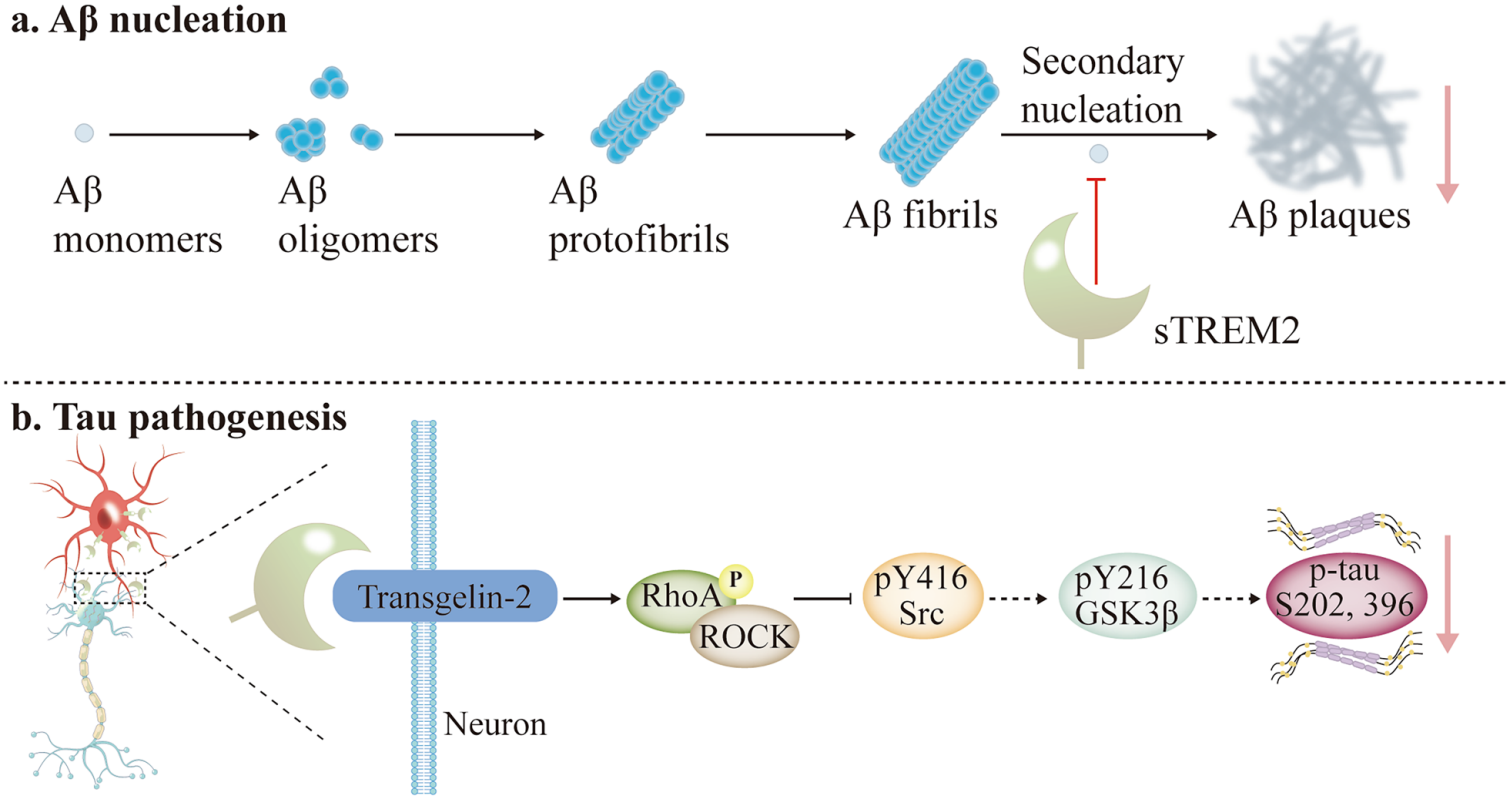
sTREM2 ameliorates Aβ and tau pathology. **a** sTREM2 binds to fibrillar Aβ, thereby inhibiting its secondary nucleation and consequently slowing Aβ fibrillization. **b** sTREM2 binds to transgelin-2, activates it, and subsequently inhibits the RhoA-ROCK-GSK3β signaling pathway. This inhibition reduces tau hyperphosphorylation, thereby improving tau pathology. sTREM2, soluble triggering receptor expressed on myeloid cells 2; Aβ, amyloid-β

As a crucial regulator of microglial function in AD pathogenesis, sTREM2 modulates neuroinflammation, amyloid plaque clearance, and Aβ pathology through multiple mechanisms. In microglia, sTREM2 enhances cell survival via the PI3K/AKT signaling [230]. Furthermore, sTREM2 promotes NF-κB phosphorylation, thereby inducing expression of inflammatory factors [230–232]. In Aβ pathology models, sTREM2 augments microglial migration toward amyloid plaques and facilitates Aβ uptake and degradation [233]. Functional mapping revealed that the amino acid segment 41–81 constitutes the minimal functional domain of sTREM2, demonstrating superior efficacy compared to full-length sTREM2 in mitigating Aβ deposition and neurotoxicity [234]. At the molecular level, nuclear magnetic resonance spectroscopy showed that sTREM2 does not interact with monomeric Aβ_40_ or Aβ_42_ [235]. Instead, sTREM2 specifically binds to Aβ fibrils and attenuates Aβ fibrillation through inhibiting secondary nucleation processes (Fig. 7a) [61, 234, 235].

In tau pathology, sTREM2 alleviates tau protein phosphorylation and cognitive deficits in mice by activating transgelin-2 (TG2) via inhibition of the RhoA-ROCK-GSK3β signaling cascade (Fig. 7b) [236]. Zhang and colleagues identified neuronally expressed TG2 as the receptor for microglia-derived sTREM2. In vitro experiments revealed that the sTREM2-TG2 binding induces RhoA phosphorylation at Ser188, thereby suppressing the RhoA-ROCK-GSK3β signaling and reducing tau phosphorylation. Importantly, sTREM2 (77–89) fragment was identified as the minimal functional sequence required for TG2 activation, which replicates the inhibitory effect on tau phosphorylation. Administration of this peptide to tau P301S transgenic mice significantly attenuated tau pathology and behavioral deficits.

sTREM2 exerts complex effects on synaptic plasticity, manifesting both neuroprotective and neurotoxic potentials. By enhancing microglial phagocytosis of Aβ, sTREM2 mitigates Aβ-induced synaptic toxicity, thereby contributing to synaptic stabilization and promoting long-term potentiation (LTP) [233]. However, some studies indicate that sTREM2 may potentially impair synaptic plasticity, and GABA_A_ receptor blockade can partially alleviate the detrimental effects on LTP [75]. Whether sTREM2 exerts these effects through direct GABA_A_ receptor activation or via modulation of GABAergic transmission to disinhibit neuronal excitability remains incompletely understood [75]. Further research is warranted to clarify the precise molecular mechanisms involved in the context-dependent regulation of synaptic function by sTREM2 during AD pathogenesis.

## TREM2 variants in AD

Genetic variants in the *TREM2* gene are associated with a variety of neurodegenerative diseases. Homozygous variants in *TREM2* are linked to the onset of Nasu-Hakola disease (NHD), a rare disorder characterized by early-onset dementia, demyelination, and bone cysts [237–240]. It is noteworthy that certain patients harboring specific homozygous mutations in the *TREM2* gene, including Q33X, Y38C and T66M, present with clinical manifestations resembling frontotemporal dementia, characterized by behavioral alterations, cognitive deterioration, and motor impairments [241]. TREM2 variants not only modulate AD susceptibility, but also exhibit functional heterogeneity in their pathophysiological impacts.

### R47H variant

R47H is the most extensively studied *TREM2* variant (rs75932628), which is a point mutation from CGC to CAC, resulting in an amino acid substitution at position 47 from arginine to histidine [10, 11]. It is worth noting that the R47H variant is in the coding region of the protein, which is quite rare for GWAS-associated SNPs [10, 11]. Although this variant has minimal effects on the TREM2 transcript level in human studies, it is associated with AD pathology, particularly involved in neuroinflammation and neurodegenerative processes [19, 242]. Compared to the wild-type TREM2, the R47H variant causes more pronounced neuropathological alterations, including reduced microglial clustering around amyloid plaques and elevated pathological tau protein levels, which may result from impairment of TREM2-mediated microglial function and subsequent exacerbation of neuroinflammation [94, 243–245].

The expression levels of TREM2 R47H variant vary significantly between mice and humans. In human carriers, TREM2 expression levels do not differ significantly from non-carriers [242], whereas in murine models, R47H expression is markedly reduced compared to wild-type [246]. Mechanistic studies revealed that the R47H variant activates a cryptic splice site in mice, causing partial exon 2 deletion, premature termination codon generation, and subsequent reduction in TREM2 mRNA/protein levels [247].

In vitro studies have demonstrated that transcriptomic alterations induced by the R47H variant substantially overlap with those observed in complete* TREM2* KO [248]. The R47H variant also modulates the expression of genes related with immune response and metabolism [249–251] and disrupts the protein structure of TREM2, particularly within the extracellular ligand domain, thereby diminishing its stability [252–254] and ligand binding affinity [37, 45, 46, 255] compared to wild type. These structural and functional alterations result in microglial dysfunction, including decreased responsiveness to Aβ plaques, thereby accelerating AD pathology progression [246, 256–258]. Furthermore, the R47H variant alters the microglial exosome secretion rates and cargo composition compared to wild-type TREM2, potentially compromising intercellular communication and neuroprotective roles of TREM2 [259]. Notably, although a recent in vitro study revealed increased cholesteryl ester levels in microglia expressing the *TREM2* R47H variant after myelin treatment [117], paradoxically, an in vivo study reported reductions of lipid droplet accumulation [260].

The R47H variant plays a complex role in both Aβ and tau pathology in various ways. In Aβ pathology, this variant disrupts Aβ oligomer clearance by impairing their binding and disaggregation, while promoting Aβ fibril formation and enhancing neurotoxicity [60, 261]. Additionally, R47H disrupts neuronal network homeostasis by elevating excitatory activity, reducing the inhibitory signal transmission [179, 262, 263], and enhancing synaptic phagocytosis, which collectively drive synaptic loss and elevate AD risk [264, 265]. The role of R47H variant in tau pathology remains controversial. In the PS19 tauopathy mouse model, homozygous R47H mutation demonstrates neuroprotective effects [266], whereas heterozygous mutation worsens spatial memory deficits [267]. A plausible explanation for this divergence is that the homozygous R47H mutation may suppress microglial activation and neuroinflammatory responses, thereby mitigating synaptic pruning and protecting against neurodegeneration and synaptic loss within the tau pathological framework [266]. Conversely, the heterozygous R47H mutation augments inflammatory reactions through activation of the AKT signaling, thereby exacerbating the tau-mediated spatial memory deficits [267]. These differential impacts of homozygous versus heterozygous mutations on physiological outcomes may resemble the disparities observed between TREM2 haploinsufficiency and complete KO.

### H157Y variant

The H157Y variant accelerates the shedding of the TREM2 extracellular domain by enhancing cleavage at the H157-S158 peptide bond, which affects the structure and function of TREM2 [46, 71, 72, 268]. In human studies, individuals carrying the H157Y variant have significantly higher levels of CSF sTREM2 [209]. In vivo and in vitro studies have demonstrated differential modulation of microglial cell function by the H157Y variant across experimental models [269, 270]. Specifically, in 5 × FAD mice, the H157Y variant decreased expression of neuroinflammation-related genes, suggesting neuroprotective potential [270]. Conversely, in the LPS-induced cellular inflammatory model, the H157Y variant enhances release of inflammatory cytokines [269]. These discrepancies likely stem from different pathophysiological mechanisms inherent to each model, necessitating further research to clarify their precise biological roles.

Future research could delve deeper into how the H157Y variant regulates the metabolism and clearance mechanisms of Aβ, which may influence the pathological progression of AD. For instance, in a cohort of Hong Kong Chinese AD patients, a certain correlation has been observed between the H157Y variant and the Aβ_42_/Aβ_40_ ratio in blood, although the specific molecular mechanisms underlying this association and the clinical significance remain to be further elucidated through larger-scale studies [271]. In vivo experimental models have demonstrated that the H157Y variant significantly promotes sTREM2 generation and accelerates Aβ clearance, effectively reducing pathological Aβ accumulation [270]. One potential mechanism is that sTREM2 inhibits Aβ aggregation and promotes its clearance in vitro, thereby potentially mediating the protective effects of the H157Y variant during Aβ clearance [233, 235].

### Other variants

The D87N variant and other TREM2 variants affect the structural stability, maturation, cell surface expression, and function of TREM2 through distinct mechanisms, thereby increasing AD risk (Table 3). The D87N substitution disrupts the D87-R76 salt bridge, reducing the structural stability of TREM2 and impeding its oligomerization/multimerization [253, 255]. By contrast, the Y38C, T66M, V126G, and W50C variants cause defective glycosylation that impairs TREM2 maturation in the Golgi apparatus. This disrupts the anterograde transport from the endoplasmic reticulum to the plasma membrane via the Golgi apparatus, leading to intracellular accumulation of immature TREM2 isoforms [37, 70, 71, 272, 273] and diminished cell surface expression [37, 71, 274–276]. The R62H variant destabilizes the immunoglobulin domain of TREM2, particularly the ligand-binding region [277], resulting in reduced ligand-binding affinity and impaired signal transduction [37, 45, 46, 60]. At the cellular level, D87N, Y38C, T66M, and W50C variants compromise TREM2 function by decreasing the ligand-binding capacity, attenuating microglial responses to inflammatory stimuli, and impairing the phagocytic activity [45, 172, 278]. The T66M variant causes an overall decline in brain metabolism in mice compared to wild-type mice [279]. The Y38C variant causes impaired myelination and myelin maintenance, a phenotype potentially linked to altered expression of myelin-related genes [280]. The Q33X variant introduces an early stop codon, thereby resulting in no TREM2 protein product. Induced pluripotent stem cell (iPSC)-derived microglia generated from Nasu-Hakola disease patients carrying the Q33X variant exhibit lysosomal dysfunction (characterized by reduced lysosomal acidification, impaired proteolysis, and accumulation of multivesicular body contents), accompanied by reduced lipid droplets and abnormal cholesterol metabolism [281]. Notably, microglia harboring the R47H and T66M variants exhibit marked deficits in clearing neuronal pathological tau aggregates through tunneling nanotubes [282].

Future research is still needed to deepen the current understanding of *TREM2* variants. First, a recent study found that the *TREM2* variant carriers show less impaired visuospatial functioning than non-carriers with biomarker-confirmed symptomatic AD. However, the mechanistic basis for this functional preservation remains unresolved [283]. Second, T96K variant carriers exhibit more pronounced memory and language deficits despite lower CSF levels of Aβ_42_, p-tau_181_ and t-tau, indicating potential involvement of distinct pathological pathways [283]. Finally, in atypical AD cases with *TREM2* variants, researchers observed aberrant NFT distribution patterns that lack clear association with Aβ deposition or neuritic plaque density [284]. Concurrently, microglial activation in these cases correlates positively with NFT density but not with the Aβ burden [284]. Collectively, these observations imply that TREM2 variants may modulate microglial function through tau-specific mechanisms in atypical AD, thereby indirectly influencing disease progression rather than directly affecting Aβ pathology.

## TREM2-targeting therapeutics

TREM2 expressed on microglial cells plays a critical role in AD pathological progression. This discovery has stimulated intensive research endeavors, culminating in the development of TREM2-targeting therapeutic strategies [285, 286]. In the following, we will review recent advancements in TREM2-based therapeutic interventions.

### Therapeutics enhancing TREM2 signaling

**Fig. 8:**
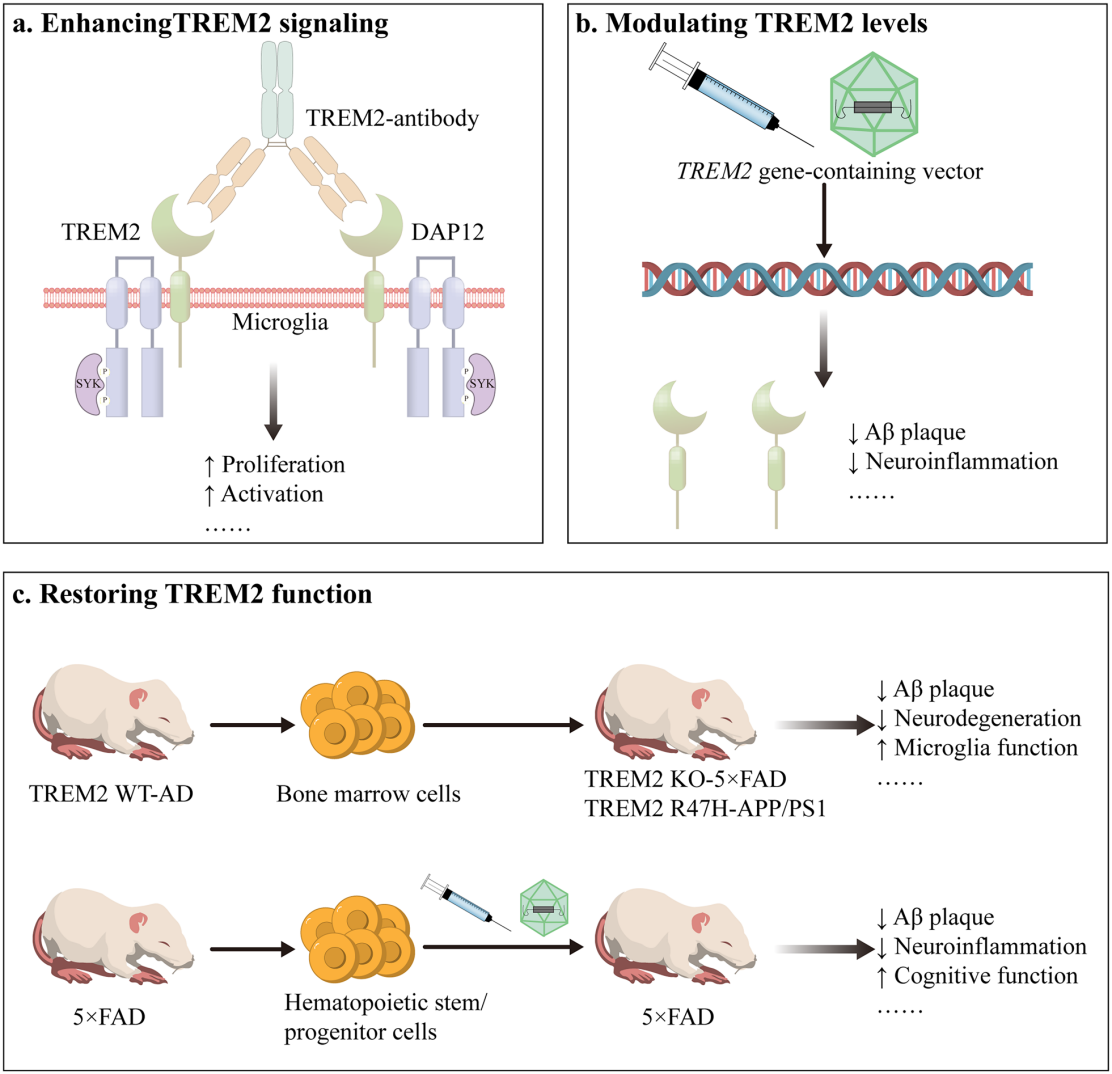
TREM2-related therapies in AD. **a** The TREM2 antibody agonist boosts TREM2 signaling, promoting microglia proliferation and activation. **b** Injection of TREM2 gene-delivering vectors (e.g., lentiviral particles, biomimetic nanovesicles) to induce TREM2 overexpression for therapeutic modulation. **c** Restoring TREM2 function is primarily achieved through two approaches. The first involves transplanting bone marrow cells from TREM2 WT-AD mice into either TREM2 KO-AD mice or TREM2 R47H mutant AD mice. The second method entails extracting embryonic stem cells from donor 5 × FAD mice, infecting them with a lentivirus carrying the *TREM2* gene, and subsequently transplanting these modified cells into recipient 5 × FAD mice. TREM2, triggering receptor expressed on myeloid cells 2; DAP12, DNAX-activating protein of 12; Aβ, amyloid-β; WT, wild type; AD, Alzheimer’s disease; KO, knockout

Agonistic anti-TREM2 antibodies have been developed to address AD through disease-modifying strategies, specifically targeting amyloid deposition and associated neuropathological alterations (Fig. 8a). These antibodies enhance TREM2 signal transduction by binding to its extracellular domain [287–296]. Key mechanisms include: (1) inhibiting TREM2 ectodomain shedding to elevate cell-surface TREM2 levels, and (2) promoting TREM2 receptor clustering to amplify the downstream signaling. Notably, these antibodies activate the TREM2-DAP12 pathway, particularly enhancing SYK phosphorylation.

Agonistic anti-TREM2 antibodies have demonstrated efficacy in ameliorating AD pathology. They promote microglial proliferation and differentiation, and enhance phagocytic activity and functional capacity of microglia [289, 291, 293–296]. Notably, agonists including 4D9, AL002a, and Ab-T1 trigger microglial phenotypic conversion to the DAM state and enhance targeted migration toward amyloid plaques [287, 290, 291, 294, 295, 297]. Agonistic TREM2 antibodies, such as 4D9, AL002c, AL002a, Ab-T1, and Ab18, can effectively attenuate amyloid deposition [287, 289, 291, 294], while Ab18 specifically alleviates tau pathology and neuroinflammatory responses [295]. AL002c, AL002a, Ab-T1, and Ab18 significantly improved cognitive function of AD mouse models, compared to untreated controls [289–291, 295]. Furthermore, the newly developed fully human anti-TREM2 antibody, M07-TFN, holds promise in mitigating the risk of immunogenicity when compared to previously investigated humanized antibodies [298]. Notably, van Lengerich et al. utilized antibody engineering technology to fuse the 4D9 antibody with the binding domain of the transferrin receptor, resulting in the construction of an innovative antibody variant ATV:4D9 [296]. This modification significantly increases the concentration and distribution of the antibody in brain tissue, thereby enhancing its therapeutic efficacy within the central nervous system [296]. Current findings for TREM2-targeting therapies derive primarily from preclinical AD models, underscoring the need for expanded phase II/III clinical trials with prolonged follow up to validate therapeutic translation [299].

Recent studies highlight natural compounds as potential modulators of TREM2 signaling to combat AD-related neuroinflammation. Hecubine, a plant alkaloid, directly activates TREM2 and reduces LPS-induced neuroinflammation [300]. The Chinese herb pyrolae herba demonstrates dual benefits: it suppresses hippocampal inflammation and improves cognitive deficits in LPS-treated mice through regulation of the TREM2 pathway [301]. Similarly, the monoterpene indole alkaloid dehydroervatamine acts as a TREM2 agonist to mitigate neuroinflammation [302]. In order to screen for more candidates with the potential to modulate TREM2 activity, researchers have successfully developed a robust TREM2 crystallization platform, laying a solid foundation for structure-based screening efforts [303]. Through methods such as molecular dynamics simulations, the importance of the CDR1 and CDR2 loops of TREM2 in ligand binding has been discovered [304–306]. Meanwhile, utilizing advanced approaches including virtual screening, molecular docking, and molecular dynamics simulations, researchers have identified multiple drug candidates, including carpipramine, clocapramine, pimozide, and varenicline [307–309]. In addition, the development of novel and efficient liposomal agonists, which mimic endogenous ligands and efficiently activate TREM2, has opened new avenues for TREM2-targeting drug design while demonstrating significant therapeutic potential in neuroinflammation, particularly in AD [310].

### Therapeutics modulating TREM2 levels

Exogenous intervention to modulate TREM2 levels can be used to ameliorate AD pathology (Fig. 8b). Various therapeutic methods and molecules, such as electroacupuncture therapy, CD33, thyroid hormone, cyclic GMP-AMP synthase, and rapamycin, have demonstrated efficacy in modulating AD progression via TREM2 regulation [311–319]. Notably, there is a complex interplay between CD33 and TREM2. Studies on human monocytes have revealed that genetic variants within the *CD33* locus associated with AD risk can lead to elevated CD33 expression, accompanied by increased TREM2 expression on the cell surface [311]. However, in the 5 × FAD mouse model, deletion of CD33 did not directly alter the expression level of TREM2 [312]. This discrepancy may stem from differences in biological characteristics between species or between cell types (monocytes versus microglia) [311, 312]. A recent study indicates indirect interactions between TREM2 and CD33 via mediators such as IL33, influencing microglial cell function [320]. Two CD33 isoforms, the long protein isoform and the short isoform, have opposing effects on microglia [321]. Therefore, future research should not only explore the interaction mechanisms between the two in different models, but also address the impact of different CD33 isoforms.

Recently, researchers have successfully achieved TREM2 overexpression by intracranial injection of lentiviral vectors expressing TREM2 or by employing transgenic technology, for precise regulation of TREM2 level [151, 322]. This intervention modulates microglial inflammatory responses, reduces Aβ deposition, decreases tau protein phosphorylation levels, and effectively alleviates cognitive impairments. However, these gene therapy strategies have encountered limitations for clinical translation, such as genetic toxicity, immune responses, and inflammatory reactions. To address these limitations, recent innovations introduced microglia-targeting TREM2 delivery systems utilizing detachable albumin nanocarriers [323]. This non-invasive vectorization strategy achieves high-efficiency transfection while enhancing microglial phagocytic capacity and anti-inflammatory activity [323]. Furthermore, using biomimetic nanovesicle platforms, researchers have developed a synchronized dual-gene modulation strategy that simultaneously upregulates *TREM2* to enhance microglial function and downregulates *BACE1* to suppress amyloidogenic processing [324]. These integrated strategies represent transformative advancements toward safe, effective TREM2-based AD therapeutics.

### Therapeutics restoring TREM2 function

Restoring TREM2 function primarily refers to replacing microglia with impaired TREM2 function through bone marrow transplantation (Fig. 8c). This approach involves transplantation of microglia derived from TREM2 wild-type-AD mice into TREM2 KO-AD mice or TREM2 R47H-AD mice via bone marrow transplantation to restore microglial function and ameliorate AD pathology [325, 326], or transplantation of hematopoietic stem cells transduced with a lentivirus overexpressing TREM2 into AD mice, leading to decreased Aβ deposition and reduced neuroinflammation [327]. However, both approaches require preconditioning regimens, such as radiation or chemotherapy, to eliminate bone marrow in recipients and establish a niche for donor stem cell engraftment [325–327]. This process frequently leads to brain injury, including blood–brain barrier disruption and induction of neuroinflammation. To address these limitations, a landmark study successfully introduced the G795A substitution in human colony-stimulating factor 1 receptor (CSF1R) using a gene-editing technique, rendering microglia resistant to depletion mediated by PLX3397/PLX5622 [328]. This enables non-toxic replacement of endogenous microglia with engineered progenitors, achieving > 90% microglial chimerism in neonatal/adult mice without irradiation [328]. Engrafted G795A-microglia persist after drug withdrawal while maintaining normal inflammatory responses [328]. In addition, Zhong and colleagues proposed a tricyclic microglial depletion transplantation strategy, utilizing the CSF1R inhibitor PLX3397 for three cycles of microglial depletion without the need for radiation or chemotherapy, thereby avoiding these potential neurotoxic effects and providing a safer and more feasible approach for microglial transplantation [329].

Finally, in vitro studies have demonstrated that the administration of a PPARγ agonist in iPSC-derived microglia harboring the *TREM2* R47H variant can enhance mitochondrial respiratory function and glycolytic capacity in microglia, as well as partially restore microglial dysfunction caused by the *TREM2* R47H variant, such as the phagocytic capacity for Aβ [257]. Supplementing iPSC-derived microglia harboring the R47H variant with specific metabolites, such as citrate and succinate, can effectively restore their metabolic dysfunction [330]. This finding suggests that TREM2-associated metabolic impairments might be mitigated through targeted metabolic intervention strategies.

### Considerations for TREM2 therapy

While agonistic TREM2 antibodies have shown promise in certain studies, their effects on AD pathology remain inconsistent, with some reports demonstrating neutral or even detrimental outcomes. For example, the agonists T2AB, AL002c, and Para.09 failed to significantly alter Aβ deposition in murine models, as evidenced by unchanged levels of both soluble and insoluble Aβ species [289, 293, 331]. Notably, chronic administration of AL002a not only failed to attenuate Aβ accumulation but also exacerbated Aβ-associated tau pathology [297]. Similarly, Para.09 administration in AD and demyelinating injury models impaired neurodegenerative recovery rather than promoting it [331]. These findings align with earlier animal studies. For example, Dhandapani and colleagues demonstrated that the genetically reduced TREM2 ectodomain shedding, despite maintenance of stable TREM2 surface expression, intensified neuroinflammation induced by Aβ pathology [332].

On the one hand, the inconsistency of these research results may partly stem from the time-dependent nature of the AD pathological process; that is, TREM2 may have different mechanisms of action at various stages of AD [333, 334]. In the early stage of AD, TREM2 facilitates the clearance of Aβ seeds by modulating microglial functions, thereby effectively limiting amyloid plaque formation (Fig. 4a) [141, 168, 176]. When the disease progresses to the middle stage of AD, and when Aβ deposition becomes the main characteristic of the pathological process, the up-regulation of TREM2 enhances the phagocytic function of microglial cells for Aβ. However, as the disease progresses to the late stage of AD, the expression levels of Aβ-binding receptors on the surface of microglial cells gradually decrease, leading to gradual decline of their phagocytic function for Aβ [335]. Even with TREM2 overexpression, this functional decline cannot be reversed [335–337]. Therefore, we speculate that in the late stage of AD, restoring the function of TREM2 in microglial cells through cell transplantation technology may be a more viable therapeutic strategy. However, the validity of this hypothesis still needs to be validated by future research.

On the other hand, detrimental effects linked to TREM2 agonist therapy likely originate from TREM2 overactivation. The variants D87N and T96K not only increase AD onset risk but also demonstrate enhanced ligand activation capacity [46]. Such findings suggest that the variant-associated TREM2 hyperactivation may accelerate AD progression by intensifying microglial receptor activity, which elevates synaptic phagocytosis and contributes to cognitive decline [338]. Conversely, moderate suppression of TREM2 function prevents excessive synaptic pruning while maintaining microglial responsiveness, thereby preserving cognitive integrity. Notably, TREM2 KO models exhibit robust neuroprotection in tauopathies, achieving reduced microglial overactivation, alleviation of neuroinflammation, and inhibition of neurodegeneration compared to wild type [158, 339]. These observations suggest timely TREM2 modulation as a therapeutic strategy. Experimental models with advanced Aβ pathology show that acute *TREM2* KO decelerates plaque deposition and lowers phosphorylated tau levels in the short term [340]; however, long-term efficacy and safety profiles require further investigation [340]. Emerging research proposes dose-dependent TREM2 regulation in human microglia through RNAase-H-active antisense oligonucleotides, which cleave target mRNA sequences to achieve precise control of expression [341, 342]. Such innovations complement ongoing efforts to develop optimized TREM2-targeting antibodies that balance therapeutic efficacy with physiological safety [343].

For assessment of drug activity through pharmacodynamics of biomarkers and determination of the optimal clinical doses, rigorous assessment of TREM2 expression and activation is essential [276, 344, 345]. Several approaches have been validated to achieve this. For instance, copper-64-labeled TREM2-specific PET tracers enable direct visualization of microglial TREM2 expression levels and have demonstrated high sensitivity/specificity in evaluating TREM2 activation in AD mouse models [346]. Additionally, CSF concentrations of sTREM2 and chitinase-3-like protein 1 correlate strongly with activation status of microglial TREM2, providing critical biomarkers for evaluating TREM2 agonist efficacy [347].

In Tg-SwDI mice susceptible to cerebral amyloid angiopathy, *TREM2* KO led to significantly reduced severity of cerebral amyloid angiopathy compared to wild type, despite increased overall amyloid deposition [348]. This observation emphasizes the multifaceted role of TREM2 in regulating distinct components of amyloid pathology. Therefore, rigorous assessment of potential side effects is needed during development of TREM2-targeted therapies. The occurrence of adverse events linked to anti-Aβ immunotherapy, such as amyloid-related imaging abnormalities, further underscores the critical need for prioritizing safety evaluations in TREM2-based treatment approaches [349]. The value of personalized strategies in designing TREM2 therapeutic regimens also warrants careful consideration [350, 351].

## Recommendations for future research

### Roles of TREM2 in physiological aging

Aging is a complex biological process characterized by gradual declines of molecular and physiological functions, significantly increasing the susceptibility to diseases, particularly neurodegenerative disorders such as AD [352]. Current research generally agrees that aging involves a series of molecular-level changes, including genomic instability, telomere shortening, alterations in epigenetic modifications, loss of proteostasis, cellular senescence, and stem cell exhaustion [353]. Aged glial cells also exhibit a senescence-associated secretory phenotype, and the inflammatory factors they secrete can disrupt the neural environment, leading to neuroinflammation and neurodegenerative changes, thereby affecting neural signal transmission and synaptic function and promoting the pathogenesis of neurodegenerative diseases [352–354].

TREM2 plays a complex and crucial role during physiological aging [355]. In C57BL/6 mice, TREM2 expression rises with age, peaks at 9 months, and declines in old age, particularly at ≥ 18 months [356–358]. In young mice, TREM2 regulates neuronal survival and synapse density [356]. However, in aged mice, TREM2 may impair nervous system function, primarily affecting microglial function, neuronal survival, and synaptic plasticity [356]. Specifically, *TREM2* KO mice show decreases of age-related neuronal loss, microglial density, oxidative stress, and complement activation compared to wild-type mice, protecting neurons from damage [359]. Furthermore, aged *TREM2* KO mice demonstrated improvement of cognitive function and hippocampal LTP, as well as increased dendritic spine density and postsynaptic protein expression compared to wild-type mice, thereby resisting cognitive and synaptic impairments [357]. These findings contrast with the neuroprotective role of TREM2 in AD, highlighting its context-dependent functions in physiological aging and pathological states.

Studies using single-cell RNA sequencing have demonstrated significant transcriptional differences between TREM2-expressing microglia in normal aging and DAM in AD (Table 2) [106, 360]. These studies revealed two novel states of microglia: white matter-associated microglia and senescent microglia [106, 360]. The white matter-associated microglia specifically localize to white matter regions in a TREM2-dependent but APOE-independent manner and participate in the phagocytosis of damaged myelin [360]. Notably, senescent microglia exhibit distinct characteristics, characterized by high expression of TREM2 and multiple senescence markers, along with a protein and transcriptomic expression profile that differs from that of DAM [106]. Spatially, these senescent microglia are not confined to the periphery of amyloid plaques [106]. As senescent microglia accumulate in the aging brain, they trigger chronic brain inflammation through secretion of pro-inflammatory factors, potentially exacerbating neurodegenerative processes and impairing neural network function [106]. The extent of their accumulation is closely correlated with cognitive decline, and elimination of these senescent microglia significantly improves cognitive function. This suggests that the senescent microglia play a pivotal role in neurodegenerative processes associated with aging and may therefore serve as a potential target for therapeutic intervention [106]. In summary, future research exploring the role of TREM2 in aging may provide new insights for AD research.

### Differences in TREM2 deletion and haploinsufficiency models

*TREM2* gene KO comprises two categories: haploinsufficiency and complete KO. Mice with *TREM2* haploinsufficiency demonstrate intermediate expression between wild-type mice and complete KO mice [361]. However, the AD pathological manifestations associated with *TREM2* haploinsufficiency do not follow a simple gene dosage correlation compared to complete KO.

Regarding Aβ pathology, *TREM2* haploinsufficiency mice exhibit a non-significant decrease in microglia surrounding amyloid plaques without substantial increases of Aβ plaque burden. In contrast, *TREM2* complete KO mice demonstrated more pronounced microglial depletion accompanied by significantly elevated Aβ plaque accumulation [126, 143, 361–364]. Notably, distinct patterns have been observed regarding tau pathology: *TREM2* haploinsufficiency produces more severe pathological characteristics than *TREM2* complete KO [361, 365, 366]. In the 5 × FAD model with AD-tau injection, *TREM2* haploinsufficiency mice display greater neuritic tau pathology compared to complete KO counterparts [361]. Additionally, microglial activation in hippocampal and cortical regions was more pronounced in haploinsufficiency cases [365]. Aging mice with *TREM2* haploinsufficiency further exhibit diminished microglial responsiveness to tissue injury and more severe cerebral atrophy [365].

This discrepancy may arise from the systemic effects of *TREM2* KO. First, *TREM2* haploinsufficiency may partially retain TREM2 function but is insufficient to fully suppress the development of tau pathology [365]. Second, *TREM2* haploinsufficiency disrupts normal signaling pathways, leading to imbalances in the expression of related genes, which further promote the progression of tau pathology [361, 367–370]. Conversely, while complete KO results in microglial dysfunction, the complete absence of TREM2 may trigger compensatory mechanisms that mitigate inflammatory responses [361, 365]. Given the relationship between tau pathology and cognitive function, future research is needed to delve deeper into the role of TREM2 in tau pathology [371].

Finally, *TREM2* haploinsufficiency may more closely mirror the pathological state in human AD. This is because most AD patients with *TREM2* variants carry only one mutated gene copy [54, 372]. Conversely, complete TREM2 KO may better recapitulate the pathological hallmarks of NHD [373]. Specifically, TREM2 KO mice demonstrate osteopenia (reduced bone mass), mirroring the bone lesions observed in NHD patients [374]. Additionally, these mice show a marked impairment in the clearance of damaged myelin and a significant reduction in the capacity to promote remyelination, similar to the demyelinating pathology seen in NHD [6, 280, 375]. Therefore, when investigating TREM2 function and its mechanistic roles, researchers must fully consider the differential impacts of KO strategies on experimental outcomes.

### TREM2 expression in microglia differs between species and genders

The transcriptomic profile of human AD microglia differs significantly from damage-associated microglia in murine models. Human AD microglia manifest a distinct transcriptional activation state, resembling reactive microglia phenotypes triggered by peripheral nerve injury [376]. In addition to this unique transcriptional activation state, human AD microglia also exhibit an enhanced human aging profile compared to microglia from older subjects [377], including upregulation of senescence-associated genes (such as *IL15*, *MS4A6A*, *MS4A4A*, *NME8*, and *GPR141*) and downregulation of genes with lower expression in microglia from older subjects (such as *CECR2*) [377].

TREM2 gene expression patterns and post-translational modifications differ among species. For example, CELF2 shows differential regulation of *TREM2* alternative splicing across species. In humans and green monkeys, CELF2 promotes exon 3 skipping during *TREM2* pre-mRNA processing, resulting in decreased production of full-length protein. However, this regulatory mechanism is absent in murine models [378]. Additionally, the human *TREM2* transcript contains an upstream AUG (uAUG) in its 5’ untranslated region (5’ UTR), which is not present in mice [379]. The human 5’ UTR suppresses translation initiation at the downstream AUG through uAUG-mediated attenuation, generating a truncated TREM2 isoform initiated from the uAUG.

As the expression levels of the TREM2 R47H variant exhibit significant differences between mice and humans, researchers have developed an improved R47H mouse model [242, 246, 247]. This R47H mouse model maintains normal splicing patterns and expression levels, circumvents cryptic splice-induced protein deficits, and more faithfully recapitulates human R47H biological functions  [380]. This model provides improved reliability for investigating AD pathogenesis, microglial heterogeneity, and neuroinflammatory responses to amyloid pathology.

TREM2 expression also demonstrates pronounced sex-dependent variation in murine models. In aged mice, female C57BL6/N and NuTRAP mice exhibit significantly elevated TREM2 levels compared to their male counterparts [381]. This difference may originate from differential microglial aging trajectories and neuroinflammatory biases, which modulate AD susceptibility [382, 383]. Notably, under TREM2 haploinsufficiency or complete KO conditions, female 5 × FAD mice develop significantly increased Aβ plaque burdens and tau pathology, whereas the males show minimal alterations [361]. However, it is important to note that many of the sex effects in the 5 × FAD mice may be caused by the Thy-1 driven expression of the transgenes (human *APP* and *PS1* with multiple familial AD mutations) [384]. In conclusion, these findings underscore the necessity for sex-stratified analyses in future TREM2 research to elucidate its precise role in AD pathogenesis.

## Conclusion

TREM2 plays a crucial role in AD. TREM2 exists primarily in two forms in the body: membrane-bound full-length TREM2 and soluble sTREM2. During AD pathogenesis, TREM2 mediates the migration, metabolism, and inflammatory responses of microglia and contributes to the development of distinct microglial subtypes. By interacting with proteins such as Aβ, tau, and ApoE, TREM2 modulates microglial function and neuroinflammation, thereby influencing AD progression. Specific mechanisms include Aβ phagocytosis, restriction of Aβ and tau protein propagation, and participation in synaptic clearance to protect against synaptic damage. sTREM2 is a potential AD biomarker released by microglia, with CSF level alterations closely associated with AD pathological progression. GWAS studies have revealed associations between sTREM2 levels and multiple AD risk-related modifiers, including the *MS4A* gene cluster. In AD pathology, sTREM2 reduces Aβ deposition and plaque-associated neurotoxicity while enhancing synaptic plasticity and modulating tau phosphorylation via neuronal receptor binding. TREM2 variants like R47H alter AD risk through mechanisms affecting cell surface expression, protein stability, ligand binding, and downstream signaling. Despite evidence supporting the impact of full-length TREM2, sTREM2, and their variants on AD pathogenesis, coupled with growing interest in therapies targeting TREM2, further mechanistic studies are required to elucidate the stage-specific contributions of TREM2 in AD. Such research is essential for optimizing therapeutic strategies. Additionally, investigations into the role of TREM2 during normal aging are critical for evaluating both its therapeutic potential and associated risks. Notably, the phenotypic differences between TREM2 haploinsufficiency and complete KO extend beyond simple gene dosage effects, with haploinsufficiency more closely recapitulating human AD characteristics. Species-specific variations in TREM2 expression patterns must be carefully considered in future studies. Further work is needed to clarify the distinct roles of TREM2 in AD pathophysiology and develop effective intervention strategies.

## Data Availability

Not applicable.

## Abbreviations

AD: Alzheimer’s disease
Aβ: Amyloid-β
NFTs: Neurofibrillary tangles
TREM2: Triggering receptor expressed on myeloid cells 2
GWAS: Genome-wide association studies
sTREM2: Soluble triggering receptor expressed on myeloid cells 2
NEU1: Neuraminidase 1
APOE: Apolipoprotein E
PS: Phosphatidylserine
TDP-43: TAR DNA-binding protein 43
C1q: Complement component 1q
IL-4: Interleukin-4
oAβ42: Oligomeric Aβ 42
DAP12: DNAX activation protein 12
SYK: Spleen tyrosine kinase
PI3K: Phosphatidylinositol 3-kinase
LILRB2: Leukocyte immunoglobulin-like receptor subfamily B member 2
ITIMs: Immunoreceptor tyrosine-based inhibitory motifs
CSF: Cerebrospinal fluid
MCI: Mild cognitive impairment
FDG-PET: [^18^F]fluorodeoxyglucose positron emission tomography
DAM: Disease-associated microglia
TLR4: Toll-like receptor 4
LPS: Lipopolysaccharide
KO: Knockout
TG2: Transgelin-2
LTP: Long-term potentiation
NHD: Nasu-Hakola disease
FTD: Frontotemporal dementia
CSF1R: Colony-stimulating factor 1 receptor
iPSC: Induced pluripotent stem cell
uAUG: Upstream AUG
5’ UTR: 5’ Untranslated region
